# pH-Responsive nanotubes from asymmetric cyclic peptide–polymer conjugates†

**DOI:** 10.1039/d4sc06288d

**Published:** 2024-12-17

**Authors:** Zihe Cheng, Qiao Song, Stephen C. L. Hall, Sébastien Perrier

**Affiliations:** a Department of Chemistry, University of Warwick Coventry CV4 7AL UK s.perrier@warwick.ac.uk; b Shenzhen Grubbs Institute, Southern University of Science and Technology Shenzhen 518055 China; c ISIS Neutron and Muon Source, Rutherford Appleton Laboratory Didcot OX11 0QX UK; d Warwick Medical School, University of Warwick Coventry CV4 7AL UK; e Faculty of Pharmacy and Pharmaceutical Sciences, Monash University Parkville VIC 3052 Australia

## Abstract

Self-assembling cyclic peptide nanotubes are fascinating supramolecular systems with promising potential for various applications, such as drug delivery, transmembrane ionic channels, and artificial light-harvesting systems. In this study, we present novel pH-responsive nanotubes based on asymmetric cyclic peptide–polymer conjugates. The pH response is introduced by a tertiary amine-based polymer, poly(dimethylamino ethyl methacrylate) (pDMAEMA) or poly(diethylamino ethyl methacrylate) (pDEAEMA) which is protonated at low pH. The self-assembling behaviour of their corresponding conjugates is investigated using different scattering and spectroscopy techniques. Compared to conjugates with hydrophilic polymeric corona, the introduction of hydrophobic polymer chains on the periphery of the cyclic peptides can prevent water molecules from penetrating through to the peptide rings, allowing the construction of hydrogen bonding interactions between cyclic peptides to form longer nanotubes. The switching between assembly and non-assembly is triggered by the change in the surrounding environmental pH, which process is controlled by the coordination between hydrophobic interactions and electrostatic repulsions. Due to the different hydrophobicity of these two polymers, the self-assembly of their corresponding conjugates varies extensively. We first demonstrate this evolution in detail and describe the relationship between the self-assembly and the inherent properties of grafted polymers, such as polymer compositions, the protonation degree of the responsive polymers and the polymer molecular weight in solutions.

## Abstract 2

We report a novel supramolecular system based on asymmetric cyclic peptide–polymer cyclic peptides. Varying protonation degrees of pH-responsive polymers can switch the assembly/non-assembly states of these nanotubes in aqueous solutions.

## Graphical abstract

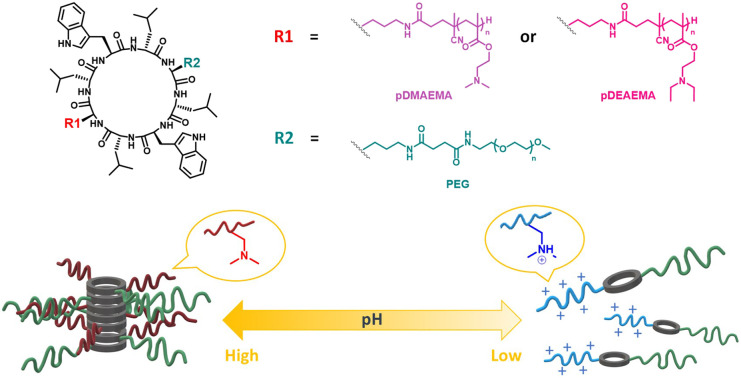

## 1 Introduction

Recently, organic nanotubes have gained considerable attention due to their various applications such as drug delivery,^1,2^ biosensors,^3^ catalysts^4^ and so on. One of the common strategies for the fabrication of organic nanotubes is supramolecular self-assembly. This method allows the self-organisation of molecular building blocks into tubular conformations.^5^ In nature, a variety of nanotubular structures formed by supramolecular assembly can be designed from diverse biomolecules like peptides,^6^ proteins,^7,8^ carbohydrates^9^ and lipids.^10^ One typical example is a natural antibiotic gramicidin A, whose linear pentadecapeptide chain can coil into helical conformation and associate head-to-head to form hollow tubular dimers, working as an ion channel on the phospholipid bilayers.^11^ Another well-known natural self-assembly instance is the tobacco mosaic virus (TMV), which consists of over two thousand identical protein molecules, assembling into rather flat discs and then stacking into successive cylinders.^12,13^ In addition, the supramolecular structures constructed by hydrogen bonds are generally remarkably stronger than those formed by other supramolecular interactions like π–π interactions.^14^ Hydrogen bonding arrays built by the alignment of multiple hydrogen bonds have enhanced strength and directionality.^15^

Cyclic peptides with alternating d- and l-amino acids are alternative synthetic building blocks, which can assemble into tubular structures by antiparallel β-sheet hydrogen bonding interactions.^16^ To avoid the uncontrolled lateral aggregation of the cyclic peptide itself and improve the solubility and stability of assemblies, polymers can be attached for the design of cyclic peptide–polymer conjugates.^5,17,18^ Moreover, the introduction of external polymers with control size and functionality enables the construction of cyclic peptide–polymer nanotubes with well-defined characteristics and properties, further expanding their various applications such as drug delivery,^19,20^ transmembrane channel mimics^21,22^ and artificial light-harvesting system.^23^

Stimuli-responsive abilities can be introduced by using specific moieties. The generated compounds can respond to changes in the external environment such as light, pH and temperature by undergoing a modification in their structures and properties. Among various stimuli strategies, the pH-triggered system has attracted increasing attention, due to its promising potential for targeted drug therapy.^24–26^ The attachment of pH-responsive polymers such as poly(acrylic acid) (pAA),^27^ poly(2-(dimethylamino)ethyl methacrylate) (pDMAEMA)^28^ and poly(2-(diisopropylamino)ethyl methacrylate) (pDPAEMA)^29^ to design pH-sensitive cyclic peptide–polymer nanotubes has been reported before. The assembly and non-assembly of these cyclic peptide–polymer nanotubes can be reversibly controlled by tuning the pH of environmental solutions. However, these studies only focused on the design of symmetric cyclic peptide–polymer conjugates, and asymmetric cyclic peptide–polymer conjugates functionalised with two different polymers, where one of them is a pH-responsive polymer, despite presenting some exciting new opportunities, have not been reported. Danial and co-workers reported that the asymmetric cyclic peptide–polymer conjugates bearing dual functionality can assemble into either Janus nanotubes with ‘dimixed’ polymeric corona or hybrid nanotubes with ‘mixed’ polymeric corona, depending on the compatibility of the two grafted polymers.^30^ So far, only a few studies have focused on these supramolecular systems based on the self-assembling asymmetric cyclic peptide–polymer conjugates.

Herein, we designed and synthesized a library of asymmetric cyclic peptide–polymer conjugates. Two tertiary amine-based methyl methacrylate monomers DMAEMA and DEAEMA (2-(diethylamino)ethyl methacrylate) were polymerized by reversible addition–fragmentation chain transfer (RAFT) polymerisation to form the pH-responsive hydrophobic segment. A hydrophilic linear PEG was introduced to improve the solubility and stability of the conjugate structure. Their self-assembling behaviour was analysed by a combination of static light scattering (SLS), transmission electron microscopy (TEM), small angle X-ray scattering (SAXS) and small angle neutron scattering (SANS), allowing to define the self-assembling systems morphology, size, shape, and aggregation number. pDEAEMA and pDMAEMA were both synthesized with two different polymerization degrees (DPs), to study the effect of the hydrophobic chain on the self-assembly behaviour. Linear PEGs with two different molecular weights (5000 and 10 000 g mol^−1^) were used, to compare the effect of the length of the hydrophilic chain. The assembled structures, with varying protonation degrees of the pH-sensitive polymer chains, were assessed at different pHs.

## 2 Results and discussion

### 2.1. Synthesis of asymmetric cyclic peptide–polymer conjugates

In order to develop the asymmetric polymeric conjugate, an asymmetric cyclic peptide scaffold bearing two amine moieties was designed, whose amine groups were protected by a Boc-protecting group and a Dde-protecting group, respectively, allowing two types of polymers to attach to the cyclic peptide in an orthogonal manner. The linear octapeptides with alternating d- and l-amino acids were synthesized by solid phase peptide synthesis using Fmoc-deprotection chemistry. The cyclization of linear peptides was achieved under dilution conditions to minimize interchain interactions, followed by the deprotection of Boc-protected cyclic peptides. The obtained peptides were confirmed by mass spectrometry and ^1^H NMR (ESI, Schemes S1, S2 and Fig. S1–S3†).

The pH-responsive tertiary amine-based polymers with controlled molecular weight were synthesized by RAFT polymerisation. In order to avoid the loss of free amine groups due to the potential side reaction aminolysis between the primary amine on the cyclic peptide and the trithiocarbonate group on the synthetic RAFT polymers, the ω-end group of polymers need to be removed before conjugation.^31^ A highly efficient photo-induced strategy was carried out under mild ultraviolet irradiation using a water-soluble hydrogen donor, ethyl piperidine hypophosphite (EPHP) (ESI, Scheme S5†). By monitoring the kinetics of this reaction, the trithiocarbonate groups were fully removed within 1 h, which was confirmed by GPC and ^1^H NMR (ESI, Fig. S9†).

**Scheme 1:**
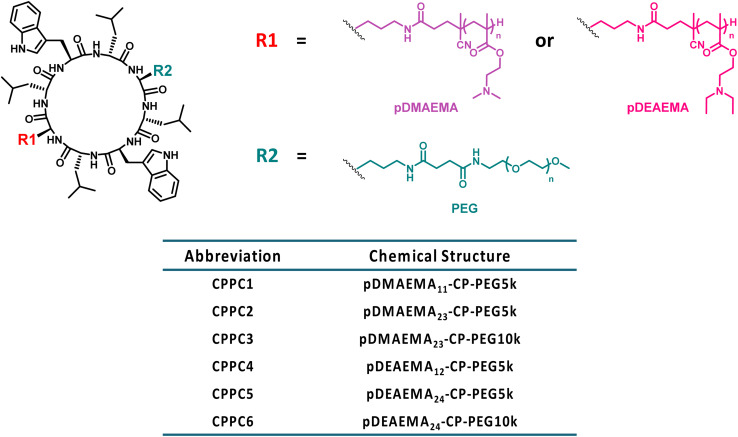
Chemical structures of asymmetric cyclic peptide–polymer conjugates based on pDMAEMA, pDEAEMA and PEG.

The hydrophilic polymer, a linear poly(ethylene glycol) (PEG) end-functionalized with a carboxylic acid group was first introduced by using HATU coupling, aiming to avoid the lateral aggregation of cyclic peptides and improve the solubility and stability of conjugate structures. Subsequently, the Dde protecting group was removed and then the responsive polymer was attached to the cyclic peptide using the same amidation method. The generated conjugates were demonstrated by HPLC, GPC and ^1^H NMR (ESI, Fig. S11†). By adjusting the DP of synthetic polymers and the molecular weight of linear PEG, a library of asymmetric cyclic peptide–polymer conjugates with dual functionality was successfully constructed under this protocol. In addition, diblock copolymers without the cyclic peptide were synthesized as control compounds (ESI, Fig. S14† and Scheme 1).

### 2.2. Polymer titration

**Fig. 1:**
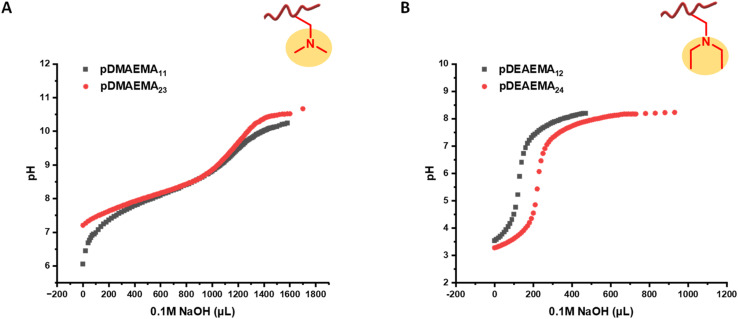
Potentiometric titration of pDMAEMA (A) and pDEAEMA (B) with two different degrees of polymerisation.

The self-assembly of cyclic peptide polymer nanotubes is mainly driven by hydrogen bonding interactions between peptide rings. Before studying the self-assembling properties of these asymmetric conjugates bearing pH-sensitive polymers such as pDMAEMA and pDEAEMA, the properties of RAFT homopolymers had to be determined first. The ionization degree (*β*) is determined by the equation below,^28^R–(CH_3_)_2_NH^+^ ⇄ R–(CH_3_)_2_N + H^+^
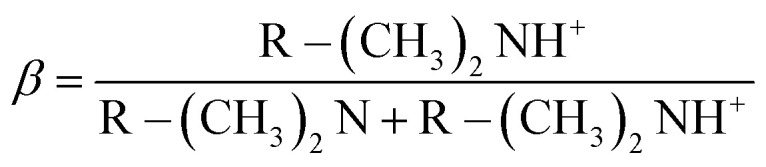
where p*K*_a_ is the pH value at which the ionization degree is equal to 0.5, which could be measured by potentiometric titration using a HI2211 pH meter (ESI†). For the DEAEMA homopolymer, the p*K*_a_ values of 8.19 and 8.30 belonged to DP11 and DP23, respectively (Fig. 1). When the pH of the surrounding solution was over the p*K*_a_ of the polymer, the hydrophobicity of polymer chains increased due to the reduced degree of protonation. However, both DEAEMA homopolymers precipitated from the aqueous solution when the pH was above 8.17 because ethyl groups are more hydrophobic than methyl groups. Although the poor solubility of the DEAEMA homopolymer, the corresponding conjugate could bear the solution pH over 13. It was interesting to study the self-assembling conformation of these asymmetric conjugates responding to different pHs.

### 2.3. Characterization of self-assembly

Each self-assembling system was investigated by changing the environmental pH.

#### 2.3.1 Static SANS measurement

SANS has been widely used in the study of cyclic peptide–polymer conjugates, including the self-assembling behaviours in different types of solvents,^32^ and responsiveness characters to different stimuli such as pH,^29^ temperature^21^ and UV light.^33^ The use of deuterated solvent provides contrast between hydrogen-rich materials and the deuterated solvent background, and can provide detailed information on the self-assembled structures.

As previously reported, the CP-PEG conjugate assemble in a core–shell cylinder structure.^32,34,35^ For the core–shell cylinder model, the first decay of the scattering data is related to an entire ‘core + shell’ dimension and the second decay represents the shell thickness.^36^ Moreover, the decay is inversely proportional to the *R*_g_ of scatterers. Two one-arm CP-PEG conjugates (CP-PEG5k and CP-PEG10k) were applied as control conjugates to study the effect of the attachment of the second polymer on the self-assembly of asymmetric conjugates. The first decay of CP-PEG10k was earlier than that of CP-PEG5k, due to the thicker shell formed by longer PEG chains (ESI, Fig. S16†). The continuous *q*^−1^ dependency extension and increased intensity observed in CP-PEG10k at small angles illustrated the presence of long nanotubes (9.7 nm), while a plateau observed in CP-PEG5k represented short nanotubes (7.2 nm).

Then, asymmetric cyclic peptide–polymer conjugates (CPPCs), including pDMAEMA_11_-CP-PEG5k (CPPC1), pDMAEMA_23_-CP-PEG5k (CPPC2), pDMAEMA_23_-CP-PEG10k (CPPC3), pDEAEMA_12_-CP-PEG5k (CPPC4), pDEAEMA_24_-CP-PEG5k (CPPC5) and pDEAEMA_24_-CP-PEG10k (CPPC6), were compared under pD3 and pD13 (pD represents pH value in deuterated solution) to study their pH response. According to our titration study, the responsive polymers were in an entire protonation state at pD3, a calculated full protonation value based on the polymerisation degree. On the other hand, pD13, far from the p*K*_a_ of two polymers, allowed for full deprotonation.

**Fig. 2:**
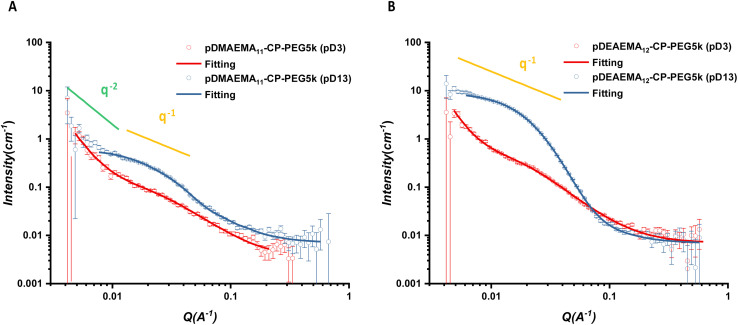
Scattering profiles and respective fits of asymmetric CPPC1 (A) and CPPC4 (B) in pD3 and pD13. The fitting model for pD3 was a Gaussian coil combined with a power law (red line) and for pD13 was a core–shell cylinder model combined with a Gaussian coil (blue line).

The scattering profiles of the six conjugates all showed remarkably different scattering signals between pD3 and pD13. A *q*^−1^ dependency observed over a *q* range from 0.00416 to 0.03 Å^−1^ represented the characteristic of individual rigid rods, illustrating asymmetric CPPCs assembled into cylindrical structures in pD13 solutions (Fig. 2 and S17–S18†). The scattered intensity of pDMAEMA conjugates exhibited a crossover from *q*^−1^ to *q*^−2^, which resulted from tube–tube interactions, further causing the formation of non-rigid large aggregates in solution.^34,37^ In contrast, this phenomenon was not observed in pDEAEMA conjugates, which may be due to the higher hydrophobicity of pDEAEMA reducing the flexibility. No *q*^−1^ dependency could be found in pD3 solutions at low *q* ranges, suggesting no aggregates could be detected and the presence of free unimeric structures.^32^

For a better structural understanding, various models were tested in the fitting process. For the assembled systems, the best model was a core–shell cylinder function. A combination of a Gaussian coil contribution at high angles could improve the model fitness. For non-assembled systems, the scattering data were fitted well with a Gaussian coil model at the intermediate and high *q* ranges. The second contribution of a power law function was applied to fit the turnover of scattering data at low *q* range. All data showed a reliable fit with these models, due to the *χ*^2^ being <5. More fitting details are included in ESI.†

**Fig. 3:**
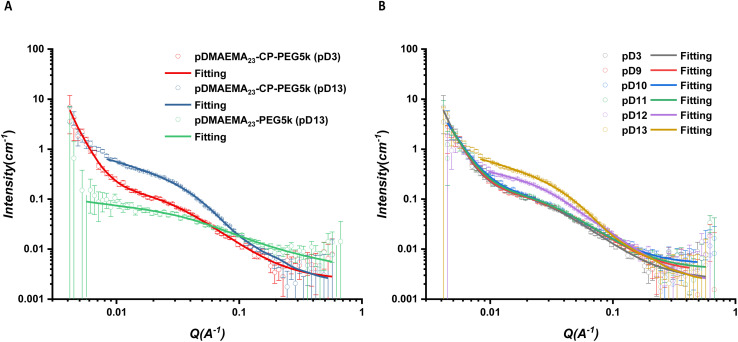
(A) Scattering profiles and respective fits of asymmetric CPPC2 and control diblock copolymer in pD3 and pD13. (B) Scattering data for CPPC2 in an aqueous solution with a range of different pDs. The non-assembled sample was fitted with a Gaussian coil model combined with a power law, while assembled one was fitted with a core–shell cylinder model combined with a Gaussian coil.

A control diblock copolymer with the same polymer chains (pDMAEMA_23_-PEG5k) was synthesized to compare to the corresponding asymmetric conjugate CPPC2 in an aqueous solution. The scattering data of the control copolymer was fitted with a Gaussian coil in the pD13 solution, since pDMAEMA had good solubility at pD13, and was not hydrophobic so that it could lead to micelle formation. However, CPPC2 assembled into cylindrical structure in the same conditions (concentration, pD and solution preparation method), highlighting aggregates driven from hydrogen bonding interactions between peptide rings (Fig. 3A).

To further study how the surrounding environment pD affected the self-assembly, CPPC2 was compared in a range of different pDs (Fig. 3B). From pD9 to pD11, the scattering data were fitted with the same Gaussian coil as that in pD3, suggesting the electrostatic repulsions on the protonation part of polymer chains could inhibit the self-assembly of asymmetric conjugates. Above pD11, the pDMAEMA chain with less charge worked as a water-soluble polymer on the peptide core, causing the conformation of cyclic peptide polymer nanotubes driven by hydrogen bonding interactions. The length of CPPC2 aggregates was 41.9 and 49.5 nm in pD12 and pD13, respectively. The higher deprotonation degree of the polymer chain was, the longer nanostructure the conjugate would form. In addition, the −2 slope of scattering intensity observed from 0.00416 to 0.008 Å^−1^ suggested the potential presence of larger aggregation resulting from interactions between each tubular structure.

**Fig. 4:**
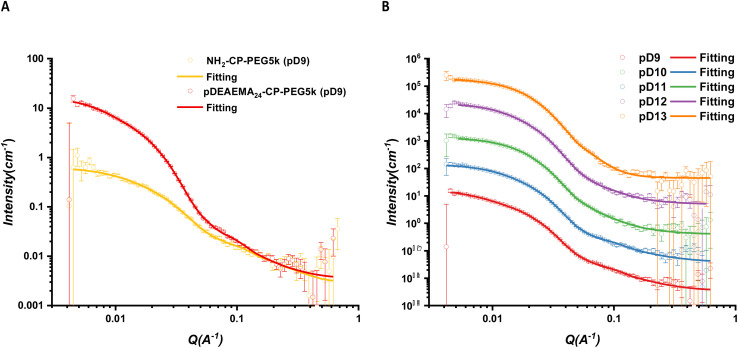
(A) Scattering profiles and respective fits of asymmetric CPPC5 and control one-arm PEG5k conjugate in pD9. (B) Scattering data for CPPC2 in an aqueous solution with different pDs. The curve separation was achieved by multiplying 10^*n*^. All scattering data were fitted with a core–shell cylinder model combined with a Gaussian coil.

At pD9, the pDEAEMA homopolymer precipitated out from solutions, while the corresponding asymmetric conjugate could remain stable in solutions and further assemble into tubular structures because the attached PEG chain provided the hydrophilicity of conjugates to stabilize aggregates. Compared with the one-arm CP-PEG5k conjugate, CPPC5 could form longer nanotubes due to the attachment of a pDEAEMA chain (50.1 nm *vs.* 7.2 nm, Fig. 4A). The deprotonation part was dehydrated simultaneously, forming the hydrophobic block between peptide cores and solvent molecules, which reduced the hydrogen bonding competition from D_2_O molecules in solutions and significantly promoted the β-sheet formation between peptide backbones.

At similar DP, pDEAEMA exhibit higher hydrophobicity than pDMAEMA, which also explained the result of the non-assembly of CPPC2 in pD9 solutions. Although an accurate p*K*_a_ of pDEAEMA was not determined, the theoretical value is slightly higher than that of pDMAEMA.^38^ Therefore, the deprotonation degree of the polymer in the same pD solution was hypothesised to be similar. The scattering profiles and fits of CPPC5 under different pD conditions were compared, see Fig. 4B. The continuous *q*^−1^ dependency extension observed in pD9 at small angles (0.00416–0.008) suggested the formation of long nanotubes, while a plateau was observed in pD13, indicating short nanotubes formed. Increasing the pD of the surrounding environment, the hydrophobicity of polymer chains was increased, witnessing a decreased trend in the length of assembled nanotubes from 50.1 nm (pD9) to 10.6 nm (pD13). As previously reported, the distance between two cyclic peptides was 0.47 nm.^16^ This phenomenon suggested that the pDEAEMA chain wrapped itself into a bigger rigid coil with increasing deprotonation degree. The increased steric hindrance on the periphery of the cyclic peptide would interfere with the hydrogen bonding interactions between peptide cores. In contrast, due to the less hydrophobicity, the entire deprotonation of DMAEMA polymers remained flexible chains in solutions. We conclude that CPPC2 forms longer nanotubes than CPPC5 in the same conditions.

**Fig. 5:**
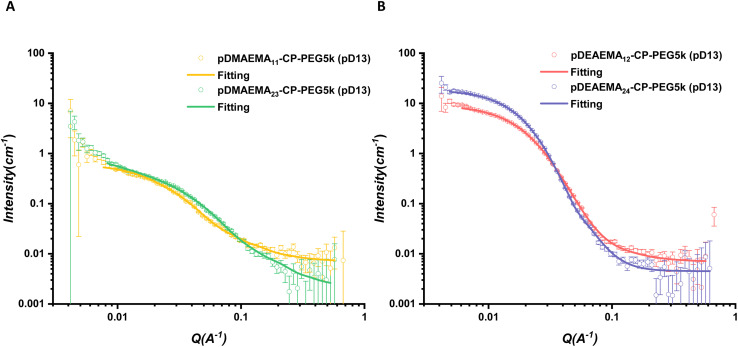
(A) Scattering profiles and respective fits of asymmetric CPPC1 and CPPC2 in pD13. (B) Scattering profiles and respective fits of asymmetric CPPC4 and CPPC5 in pD13. All scattering data were fitted with a core–shell cylinder model combined with a Gaussian coil.

Two pairs of asymmetric CPPCs were compared to study the effect of the length of hydrophobic chains on the self-assembly behaviours (Fig. 5). CPPC1 formed the cylindrical structure with a length of 20.9 nm at pD13. Doubling the DP of the pDMAEMA chain, CPPC2 formed longer nanotubes with a length of 49.5 nm (pD13), suggesting the increased hydrophobicity on the periphery of cyclic peptides benefited the formation of longer nanotubes. The same conclusion could be made from CPPC4 and CPPC5 at pD13. CPPC5 formed nanotubes with a length of 10.6 nm, which was longer than that of CPPC4 aggregates (6.6 nm). In the previous study, the attachment of two PEG chains on the cyclic peptide increases the hydrophilicity of the corona, allowing the D_2_O molecules to reach the peptide cores and join in the competition of hydrogen bonding sites, further shortening nanotubes.^32^ In this case, one hydrophobic polymer could work as a block to prevent solvent molecules from penetrating through, benefitting the construction of β-sheet hydrogen bonding interactions between cyclic peptides.

**Fig. 6:**
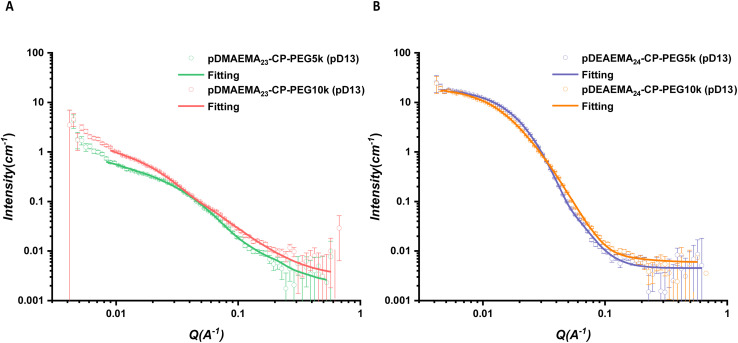
(A) Scattering profiles and respective fits of asymmetric CPPC2 and CPPC3 in pD13. (B) Scattering profiles and respective fits of asymmetric CPPC5 and CPPC6 in pD13. All scattering data were fitted with a core–shell cylinder model combined with a Gaussian coil.

In addition, two linear PEG chains with different molecular weights were introduced to study the effect of the length of hydrophilic polymers on the self-assembly of asymmetric conjugates (Fig. 6). As discussed above, CP-PEG10k formed slightly longer nanotubes than CP-PEG5k as the longer chains provide a much bulkier shield to allow peptide stacking. For the asymmetric conjugates, the length of CPPC3 nanotubes (100 nm) showed a remarkable increase compared to that of CPPC2 (49.5 nm), suggesting the longer PEG chains could mix with the DMAEMA polymer chains to increase the density of polymer corona and inhibit the interference between solvent molecules and hydrogen bonding sites. In previous work, a peptide with two PEG chains (10 000 g mol^−1^) was shown to form tubular structures with a length of around 14 nm by SANS.^39^ Keeping the same peptide but with only one 10 000 g per mol PEG chain, the attachment of a DMAEMA polymer increases the length of peptide nanotubes remarkably, to over 100 nm. This is consistent with our hypothesis and highlighted the effect of the introduction of hydrophobic polymers. Although the DEAEMA polymer with a higher hydrophobicity would tend to form a rigid coil surrounding the peptide rather than a flexible chain, longer linear PEG could also provide more shield to allow the β-sheet stacking of peptide cores. The length of CPPC6 (14.8 nm) was longer than that of CPPC5 (10.6 nm).

**Fig. 7:**
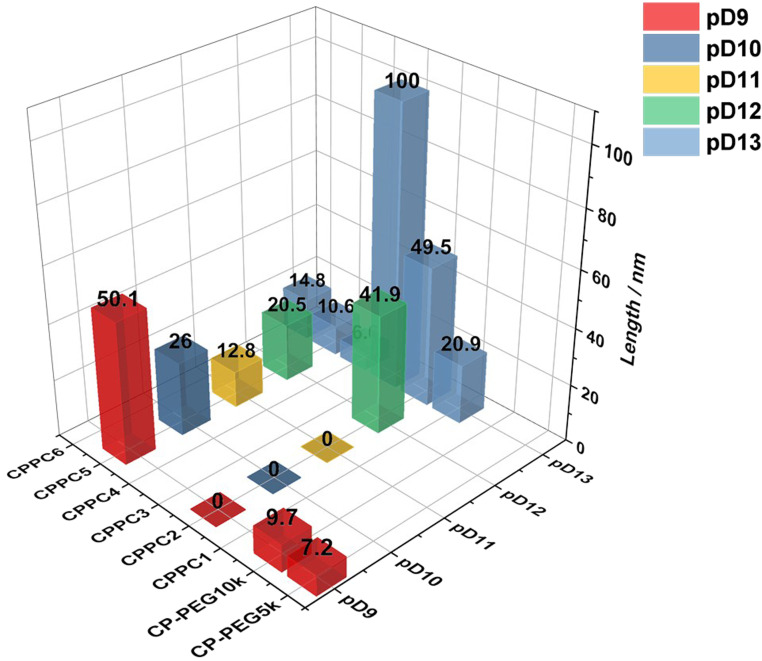
Summary of the length of self-assembling nanotubes formed by different kinds of cyclic peptide–polymer conjugates (CPPCs) in different pD conditions (0 means no assembly).

In contrast with common amphiphilic block copolymers that can self-assemble into various structures due to different block ratios, these systems were independent of the component of two polymer chains. They remained tubular structures in solutions with the increase of hydrophobic mass fraction from 31 to 48 wt%, highlighting the effect of supramolecular aggregation on the original block copolymer self-assembly. The fitted length of all assembled nanotubes under each condition is summarized in Fig. 7.

#### 2.3.2 SANS study of time-resolved measurement

**Fig. 8:**
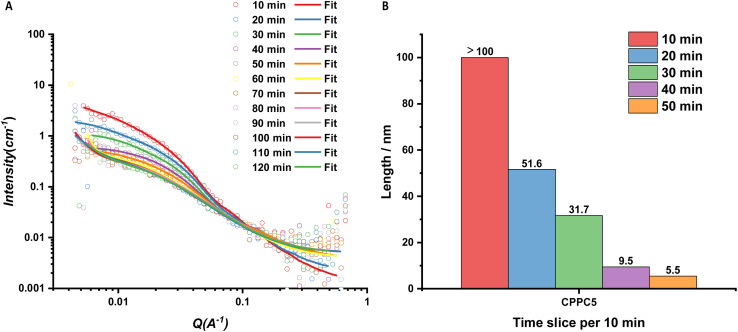
(A) Scattering profiles and respective fits of CPPC5 for the time-resolved experiment. Within 50 min, the scattering data were fitted with a core–shell cylinder model combined with a Gaussian coil. The other data were fitted with a Gaussian coil model combined with a power law. (B) Summary of the fitted length change of self-assembling nanotubes over time.

Based on the static study, pH-responsive asymmetric conjugates could form tubular structures in high pD and remain free unimers in low pD. However, how the pD of the surrounding environment affected the structural transition in solutions was unknown. Here, glucono-δ-lactone (GdL) was introduced, as its slow hydrolysis generates gluconic acid, leading to an *in situ* uniform pH change.^40,41^ CPPC5 was selected to take this time-resolved experiment under the addition of GdL. Kinetic runs and scattering data collection were taken each 10 min (Fig. 8A).

There was an apparent decrease in the scattering intensity from the Guinier region to the intermediate section. The *q*^−1^ dependency disappears over a *q* range from 0.00416 to 0.03 Å^−1^ where the form factor was determined. The fitting model was used the same one as the static data fitted. For the 60 min slice, the fitted length was smaller than 1 Å, which was not reasonable to use the core–shell cylinder model. All scattering data were fitted with the Gaussian coil model, and showed that after 1 hour of the addition of GdL, the peptide nanotube disassembles entirely.

From 10 to 50 min, there was a decreased tendency in the length, from over 100 nm to 5.5 nm (Fig. 8B). A finite value of the length of the first 10 min slice was not observed, due to the limitation of the observation window for SANS (1000 Å). For analysis, the maximum length was set at 1000 Å, while the length was over 100 nm in fact. As the pD dropped in the solution, the increasing protonation of pDEAEMA gained more chain flexibility, but the increased electrostatic repulsion led to the formation of shorter peptide nanotubes.

**Scheme 2:**
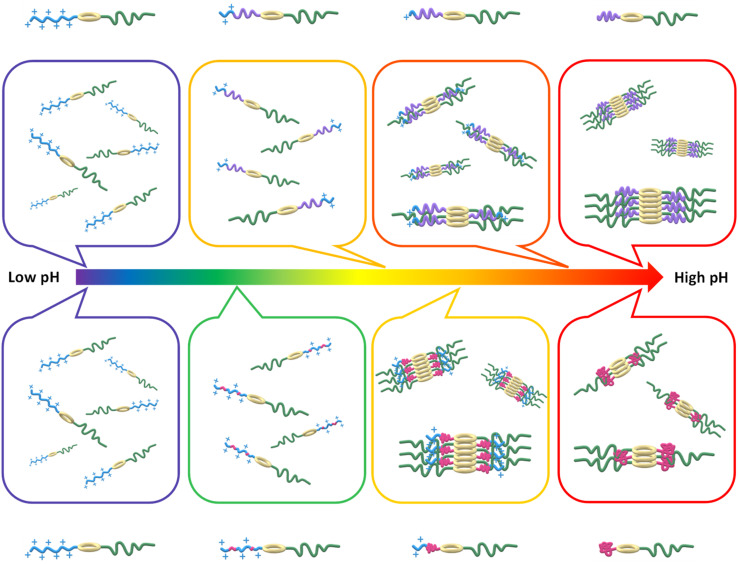
Scheme representation showing the different self-assembling evolution of pH-responsive nanotubes varying with environmental pH due to the different hydrophobicity of attached polymers (pDMAEMAs (purple) and pDEAEMAs (pink)).

The fitted length for pD9 in the static study was 50.1 nm, and the length decreased with the increase of pD value. The pD value of the first 10 min slice was lower than pD9, which is explained by the free pDEAEMA chain precipitating out from the solution when pD was above 8.17. We hypothesise that the longest nanotubes formed by the asymmetric pDEAEMA-based conjugates are due to the pDEAEMA chain still soluble in solutions with the critical protonation degree. A further decrease in pD causes competition between the hydrophobic interaction and the electrostatic repulsions. When the hydrophobic polymers cannot provide a sufficient hydrophobic shield to protect the peptide–peptide H-bonds, the electrostatic repulsions of the charged chains lead to the disassembly of the whole structure. The fitted SLD value for the polymer shell steadily approached the solvent SLD value over time, confirming the polymer corona is swollen by solvent. This experiment therefore explained the behaviour of the conjugates between pD3 and pD9, and confirmed the pH-responsive behaviour of the asymmetric conjugates is reversible in solution (Scheme 2).

#### 2.3.3 TEM study

TEM is another complementary technique to determine the morphology of the self-assembling conjugates. To improve contrast, a negative staining uranyl acetate (UOAc) was employed. The assembled nanotubes formed by conjugates could be visualized by TEM. Using ImageJ software, 100 nanotubes were randomly counted, so the distribution of length and diameter could be collected. The one-arm CP-PEG5k conjugates could self-assemble into tubular structures with around 56.8 nm in length and 4.5 nm in width, while two-arm CP-(PEG5k)_2_ conjugates formed nanotubes with around 43.5 nm in length and 3.35 nm in width (ESI, Fig. S19†). The diameter difference between these two kinds of nanotubes was small, while the length of CP-(PEG5k)_2_ nanotubes was much shorter than that of CP-PEG5k nanotubes, which was consistent with the discussion above.

**Fig. 9:**
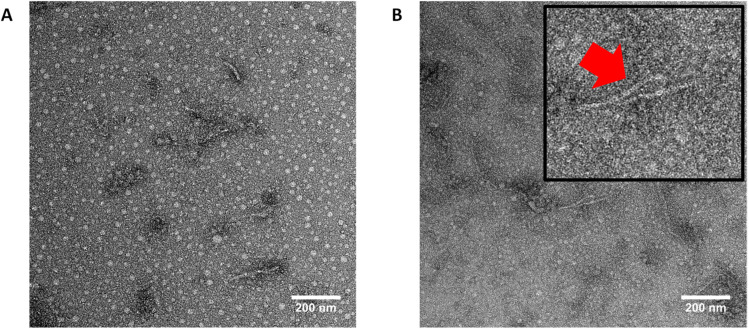
(A) TEM image of asymmetric pDEAEMA_24_-CP-PEG5k(CPPC5) in pH9 (stained with UOAc). (B) A magnified view of these asymmetric nanotubes.

The asymmetric conjugate pDEAEMA_24_-CP-PEG5k (CPPC5) was selected because this conjugate could assemble into nanotubes in pH9, as confirmed by SANS. The assemblies of CPPC5 were observed with various lengths, we could not count 100 nanotubes of CPPC5 assemblies on the grid, but the length of long nanotubes was over 100 nm (Fig. 9). We speculated some systems could disassemble during the staining process due to an acidic environment of UOAc. In addition, the white dots in the image were due to the aggregation of PEG chains, which could happen during solvent evaporation and hydrogen bonding break.

#### 2.3.4 SLS study

SLS is an excellent technique to study polymer and biomolecular materials in solutions, providing information about molecular weight, aggregation number and size of macromolecules.^42,43^

**Fig. 10:**
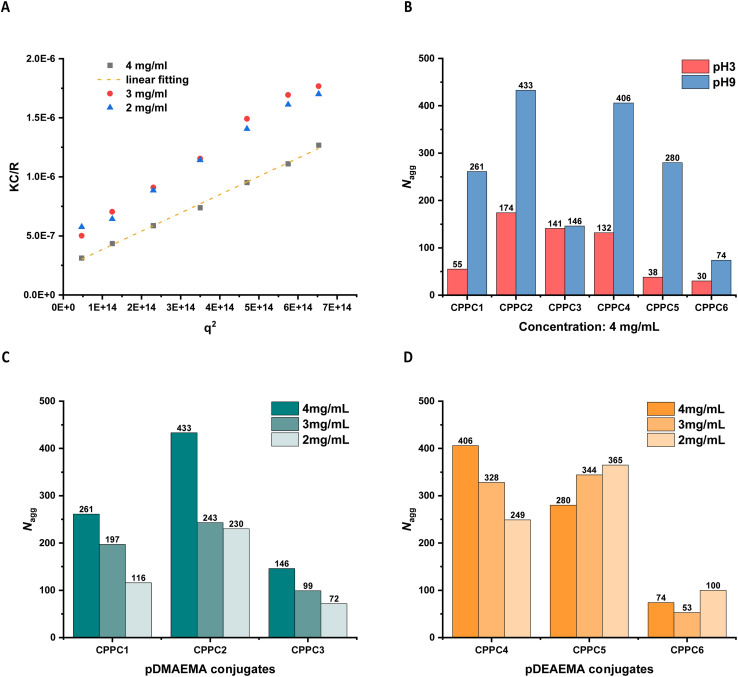
(A) Evolution of KC/R of asymmetric conjugate CPPC2 at pH9 as a function of *q*^2^ obtained by static light scattering. (B) Summary of the aggregation number for different conjugates with a concentration of 4 mg mL^−1^ at pH3 and pH9. Comparison of the aggregation number for pDMAEMA conjugates (C) and pDEAEMA conjugates (D) at pH9 (2.0–4.0 mg mL^−1^).

The large macromolecules with scattering angle dependency were analysed by the Zimm plot method. In Fig. 10A, the evolution of KC/R of asymmetric conjugate CPPC2 at pH9 was shown as a function of *q*^2^. The intercept represented the reciprocal of the molecular weight of the aggregate under different concentration conditions. Due to the known molecular weight of an unimeric conjugate, the aggregation number was obtained by dividing the aggregation molecular weight by the single unimer molecular weight. In addition, the intermolecular distance between cyclic peptides of peptide nanotubes was 0.47 nm.^16^ The length of self-assembling nanotubes was apparent by multiplying this distance with the calculated aggregation number.

Fig. 10B compared the calculated aggregation number of six different asymmetric conjugates (4 mg mL^−1^) in solutions at pH3 and pH9, suggesting the pH responsiveness was presented in most cases because of a larger aggregation number observed at pH9 while a smaller one at pH3.

As determined by SANS, CPPC2 did not form tubular structures in pD9 due to weak hydrophobicity and strong electrostatic repulsions (Fig. 3). Due to the lack of information about other conjugates, we analysed these six conjugates by SAXS to identify their assembly or non-assembly properties in pH9 solutions (ESI, Fig. S20†). All pDMAEMA conjugates failed to form nanotubes, but the increased intensity at the low *q* range observed in both SANS and SAXS confirmed the presence of large aggregation relative to intermolecular interactions, suggesting these observed aggregates belonged to the unimeric aggregation. Moreover, this aggregation behaviour was concentration-dependent (Fig. 10C). The larger number of unimers in solutions, the more aggregates formed due to the decreased intermolecular distance. Compared with CPPC1, CPPC2 with a double molecular weight of the pDMAEMA chain tended to associate with each other due to the increased hydrophobicity, leading to the construction of large aggregates. In addition, the longer PEG chain of CPPC3 would reduce the tendency of their unimeric association due to its steric effect.

In the case of pDEAEMA conjugates, CPPC4 did not form tubular assemblies due to the absence of *q*^−1^ dependency peak, so we expect it follows the same rule as the pDMAEMA conjugates. However, self-assembling nanotubes could be observed in CPPC5 and CPPC6 solutions, resulting from the increased length of the pDEAEMA chain provided sufficient hydrophobic shields to allow intermolecular hydrogen bonds formation between cyclic peptide rings. In Fig. 10D, SLS data for CPPC5 (*N*_agg_ = 365, length = 172 nm, pH9, 2 mg mL^−1^) was larger than the nanotube fitted by SANS (length = 50.1 nm, pD9, 2 mg mL^−1^), since SLS analysis provides information outside the SANS observation window (maximum 100 nm). SLS measurement confirmed the existence of long nanotubes constructed by the stacking of several short assemblies, which was consistent with TEM result above (Fig. 9).

In addition, the SLS data of CPPC6 shows much smaller assemblies that those of CPPC5, since the longer PEG corona for CPPC6 could prevent the elongated association from another single nanotube, so this data only reflected the aggregation situation of a short nanotube (*N*_agg_ = 100, length = 47 nm, 2 mg mL^−1^). Although CPPC6 had not been run by SANS at pD9 for comparison, the length of SLS was acceptable because the fitted length of a CPPC5 nanotube at pD9 was 50.1 nm (2 mg mL^−1^).

## 3 Conclusion

To conclude, we successfully designed and constructed a library of pH-responsive asymmetric cyclic peptide–polymer conjugates, which form hybrid nanotubes in aqueous solutions due to the good compatibility (miscibility) of the two grafted polymers. The introduction of the hydrophobic polymer on the periphery of cyclic peptides hinders water molecules from penetrating peptide rings, thus permitting the formation of hydrogen bonds between cyclic peptides. As the hydrophobicity of pDMAEMA and pDEAEMA vary with pH, we could engineer pH-responsive peptide nanotubes in solutions. Moreover, the hydrophobicity difference between pDMAEMA and pDEAEMA resulted in different self-assembling processes.

Initially, responsive polymers were fully protonated in solutions, so no tubular assemblies formed due to strong electrostatic repulsions. With the increase of environmental pH, the hydrophobicity of polymer chains increased. During this stage, the self-assembly was controlled by the coordination between hydrophobic interactions and electrostatic repulsions. Most importantly, we observed a change in the solubility and stability of the two polymers under the different protonation degrees, resulting in different evolutions of the self-assembling process. For the pDMAEMA-based conjugates, a higher deprotonation degree of polymers led to longer nanotubes, since the pDMAEMA chains remain flexible in solutions even if fully deprotonated. This behaviour was contrasted with the control copolymer pDMAEMA-PEG without a cyclic peptide centre, which did not form aggregates, thus highlighting the importance of peptide–peptide interactions. However, upon an increase in pH, as the pDEAEMA chains are no longer soluble, they collapse and wrap into a rigid coil near the peptide ring, although the assembly remain stable in solution due to stabilization by the hydrophilic PEG chains. When these pDEAEMA coils expand with increased pH, the steric hindrance of these coils interferes with the hydrogen bonds between cyclic peptides, thus leading to shorter nanotubes. In addition, the PEG chain improved the solubility and stability of asymmetric conjugates. The longer PEG chains provided more shielding for peptide cores, thus allowing longer nanotubes to form in solutions. Moreover, compared with the short PEG chain, a longer PEG chain could prevent lateral aggregation between single nanotubes.

Overall, we have demonstrated a general synthetic pathway for pH-responsive cyclic peptide–polymer nanotubes in aqueous solutions. By adjusting the environmental pH, the assembly and disassembly of these peptide nanotubes can be controlled by the responsive polymers switched between uncharged and charged states.

## Data availability

The data supporting this article have been included as part of the ESI.†

## Author contributions

Z. C. conducted the experimental and wrote the manuscript with the support of S. P., Q. S. and S. C. L. H. SANS measurements were performed by S. C. L. H. SANS fitting were performed by Z. C. and S. C. L. H.

## Conflicts of interest

The authors declare no competing interests.

## Supplementary Material

SC-OLF-D4SC06288D-s001
