# Supplementary material for: pH-Responsive nanotubes from asymmetric cyclic peptide–polymer conjugates
Source: Chem Sci. 2024 Dec 17;16(4):1894–906. doi: 10.1039/d4sc06288d (PMC11665818; doi:10.1039/d4sc06288d)
Supplement: SC-016-D4SC06288D-s001 [file SC-016-D4SC06288D-s001.pdf]

Supplementary Information (SI) for Chemical Science.
This journal is © The Royal Society of Chemistry 2024

Supporting Information

Materials and Characterization

Materials

Protected amino acids (Fmoc-D-Leu-OH, Fmoc-L-Lys(Boc)-OH, Fmoc-L-Lys(Dde)-OH, Fmoc-L-Trp(Boc)-OH) and 2-(6-Chloro-1-H-benzotriazole-1-yl)-1,1,3,3-tetramethylaminium hexafluorophosphate (HCTU) were purchased from Iris Biotech GmbH (Germany). Dimethyl-2,2-azobis(2-methylpropionate) (V601) was purchased from Wako Chemicals (UK). Functionalized Linear PEG polymers were purchased from Rapp Polymere (Germany). 1-[Bis(dimethylamino) methylene]-1H-1,2,3-triazolo[4,5-b] pyridinium 3-oxid hexafluorophosphate (HATU) was purchased from Alfa Aesar. 4-(4,6-dimethoxy-1,3,5-triazin-2-yl)-4-methyl-morpholinium tetrafluoroborate ($DMTMM{\cdot}BF_4$) was synthesised from 2-Chloro-4,6-dimethoxy-1,3,5-triazine (DMTMM·Cl, Sigma-Aldrich), *N*-methylmorpholine (NMM, 99%, Sigma-Aldrich) and Sodium tetrafluoroborate (Sigma-Aldrich). 2-(diethylamino)ethyl methacrylate (DEAEMA), 2-(dimethylamino)ethyl methacrylate (DMAEMA) were purchased from Sigma-Aldrich. All monomers were passed through aluminium oxide to remove the inhibitor. Spectrum™ Spectra/Por™ 6 pre-wetted standard regenerated cellulose dialysis membrane was purchased from Fisher Scientific. Deuterated hydrogen chloride and sodium hydroxide were purchased from Sigma-Aldrich, UK. 2% uranyl acetate solution was prepared by RTP, School of Life Science. Solvents were purchased from departmental suppliers, including Sigma-Aldrich, Fisher Scientific and Honeywell.

Characterization

Nuclear Magnetic Resonance Spectroscopy (NMR)

^{1}H NMR spectra were measured using either a Bruker Avance III HD 300 MHz or a Bruker Avance III HD 400 MHz NMR spectrometer. Polymers were dissolved in deuterated chloroform (*d*-$CDCl_3$) and deuterated dimethyl sulfoxide (*d*-DMSO). Peptides and conjugates were dissolved in deuterated trifluoroacetic acid (*d*-TFA). The residual solvent peaks were used as internal references.

Electron Spray Ionization Mass Spectrometry (ESI-MS)

ESI-MS was measured using an Agilent 6130B instrument with positive and negative ionization modes (800-2000). All samples were prepared in methanol and then filtered by 0.2 μm pore size PTFE membranes for analysis.

High-Performance Liquid Chromatography (HPLC)

HPLC was measured using a Shimadzu Prominence HPLC, equipped with an Agilent Technologies InfinityLab Poroshell 120 EC-C18, 4.6 x 100 mm, 4 μm, 120 Å analytical LC column. The mobile phase was acetonitrile/water. All samples were prepared in 20% acetonitrile in water with 0.04 vol% TFA and filtered by 0.45 μm pore size Nylon membranes before measurement. Detection was achieved *via* monitoring UV absorption at 280 nm and 309 nm.

Gel Permeation Chromatography (GPC)

THF GPC was measured using an Agilent Infinity II MDS instrument equipped with equipped with differential refractive index (DRI), viscometry (VS), dual angle light scatter (LS) and multiple wavelength UV detectors. Two PLgel Mixed C columns (300 x 7.5 mm) and a PLgel 5 µm guard column were included in the system calibrated using poly (methyl methacrylate) and polystyrene standards (Agilent EasiVials). The eluent is THF with 2 % TEA and 0.01 % BHT additives. All samples were prepared by dissolving in THF and then filtered by 0.2 µm pore size PTFE membranes before measurement. Exported data were processed by the Agilent GPC/SEC software.

PL50 DMF GPC was measured using an Agilent PL50 instrument equipped with differential refractive index (DRI) and UV detectors. Two PLgel M columns (300 mm × 7.5 mm) and a PolarGel 5 µm guard column were included in the system calibrated using poly (methyl methacrylate) standards (Agilent EasiVials). The eluent is DMF with 0.1 % LiBr additive. All samples were prepared by dissolving in DMF and then filtered by 0.2 µm pore size PTFE membranes before measurement. Exported data were processed by the Agilent GPC/SEC software.

Small angle neutron scattering (SANS)

SANS was measured on Larmor at the ISIS Pulsed Neutron Source (STFC Rutherford Appleton Laboratory, Didcot, UK). Conjugate samples were dissolved in a 5% DMF-d D_2O solution, with a concentration of 2 mg ml^{-1}. Solutions were placed in a 2 mm path length quartz cuvette. The scattering intensity is represented as a function of the scattering vector Q, which is related to the wavelength of the incoming beam λ and the scattering angle 2θ, by

$$Q = \frac{4\pi}{\lambda} sin\theta$$

The scattering cross-section was measured over a Q-range of 0.004 - 0.5 $Å^{-1}$ by utilizing an incident wavelength range of 0.9 - 13.3 Å. The detector is located 4.1 m from the sample and is 664 mm wide × 664 mm high with the beam in the centre of the detector. The beam size is 6 mm wide and 8 mm high. Each raw scattering data set was corrected for the detector efficiencies, sample transmission and background scattering and converted to scattering cross-section data ($\partial\Sigma/\partial\Omega$ vs. Q) using Mantid software.[1] These data were placed on an absolute scale (cm^{-1}) using the scattering from a standard sample (a solid blend of hydrogenous and perdeuterated polystyrene) in accordance with established procedures. The contrast derives from the scattering length density (SLD) difference between the sample ρ_{sample} and the solvent background ρ_{solvent}. The SLD is defined as the summation of scattering lengths from N atoms divided by occupied volumes of all atoms,

$$SLD = \frac{\sum_{i=1}^{N} bi}{Vm}$$

where b_i is the summation of the scattering length contribution of i atoms and V_m is the total volume of N atoms. For known density and chemical composition, the SLD can be calculated by the following equation,

$$SLD = \frac{\rho Na \sum_{i=1}^{N} bi}{\sum_{i=1}^{N} Mi}$$

where N_a is the Avogadro constant, ρ is the bulk density of the material and M_i is the atomic molar mass for each element. The SLD value was held constant during the fitting process.

Static light scattering (SLS)

SLS was measured using an ALV/CGS-3 Compact Gonimeter instrument equipped with a monochromatic beam of laser light under a wavelength of 632 nm at 25 °C. All conjugate samples were prepared in 5% DMF aqueous solvent with a range of concentration gradients: 2.0 - 4.0 mg/mL. Before measurement, all sample solutions were filtered by 0.45 µm pore size Nylon membranes.

Small angle X-ray scattering (SAXS)

Conjugates were dissolved in the purified water previously filtered by 0.45 µm pore size Nylon membranes and prepared to the concentration of 5 mg/mL. Solutions were loaded into borosilicate capillaries with a path length of 1 mm. SAXS was measured using a Xenocs Xeuss 2.0 diffractometer equipped with a Dectris Pilatus 1M detector positioned 5 m from the sample with a monochromatic X-ray beam of 1.54 Å wavelength, including the effective scattering vector (Q) range of 0.004 – 0.15 $Å^{-1}$. Data were collected for 4 hours at room temperature. Data were normalized to the flux of the incident direct beam to place the intensity on an absolute scale.

Transmission electron spectroscopy (TEM)

1mg/mL solution of the cyclic peptide polymer conjugate was prepared by dissolving in 5% DMF overnight and then water was pumped into the vial by a syringe pump. After the mixture, the sample vial was left on the roller overnight before measurement. 10 µL aqueous solution of conjugates was drop-cast on the TEM grid with carbon-coat. After 3 min, the excess solution was absorbed with filter paper. The sample grid was dried for 30 min and then 10 µL 2% uranyl acetate solution was dropped onto the grid for 30s. Bright field of TEM micrographs were obtained with a JEOL 2100Plus microscope operating at 200kV, equipped with a Gatan OneView IS camera.

Synthetic protocols

Peptide synthesis – NH_2-CP-Dde

Linear peptide synthesis

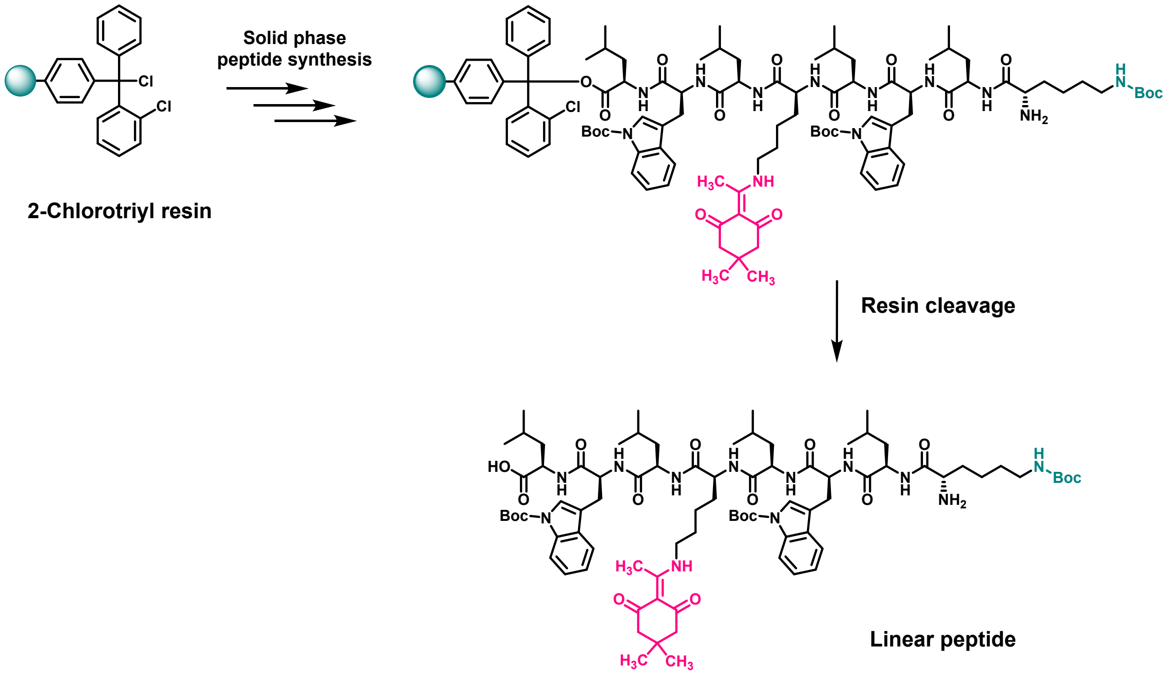

Scheme S1 Example of synthetic process of the linear peptide by solid phase peptide synthesis (SPPS).

The linear peptide with the sequence of H_2N-L-Lys(Boc)-D-Leu-L-Trp(Boc)-D-Leu-L-Lys(Dde)-D-Leu-L-Trp(Boc)-D-Leu-OH was synthesized by solid phase peptide synthesis on a Prelude Automated Peptide SynthesizerTM (Protein Technologies Inc.) using 2-chlorotrityl chloride resin as the solid support. The first Fmoc protected amino acid was coupled to the resin using DIPEA (4 eq.) in DCM, followed by capping of unreacted resin sites using a solution of MeOH:DIPEA:DCM (7:1:2, v/v/v). Deprotection of the Fmoc group of the amino acids was done using 20% piperidine in DMF. Subsequent amino acids were coupled using Fmoc-amino acids (5 eq.), HCTU (5 eq.) and NMM (10 eq.) in DMF. The linear peptide was cleaved from the resin by using 20% HFIP in DCM solution and finally dried to obtain a white solid.

Yield: 504.4 mg.

ESI-MS m/z: 1585.8 $[M+Na]^+$ (calculated: 1585.9), 1561.8 $[M-H]^-$ (calculated: 1561.9).

^{1}H NMR (400 MHz, Trifluoroacetic acid-*d*) δ(ppm): 8.26-8.05 (d, 2H, Trp), 7.77-7.20 (m, 8H, Trp), 5.27-5.03 (m, 2H, H_αTrp), 4.85-4.49 (m, 6H, H_αLeu and H_αLys), 3.85-3.62 (m, 2H, CH_2-Dde), 3.63-3.39 (m, 2H, CH-NH_2 Lys), 3.39-3.06 (m, 10H, CH_2 Trp, C**H_2**-NHBoc Lys and CO-C**H_2**-$C(CH_3)_2$), 2.15-1.18 (m, 51H), 1.19-1.09 (m, 6H, $C(CH_3)_2$ Dde), 1.09-0.62 (m, 24H, CH_3 Leu).

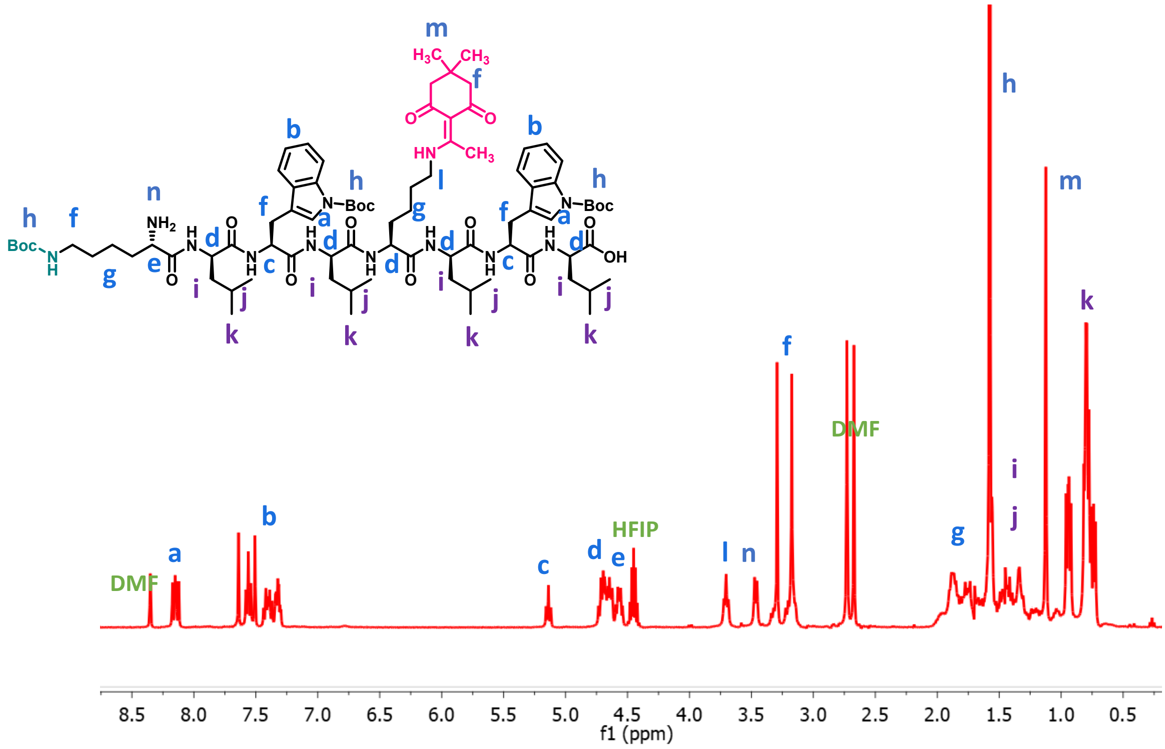

Figure S1 ^{1}H NMR spectrum of linear peptide in *d*-TFA.

Cyclization

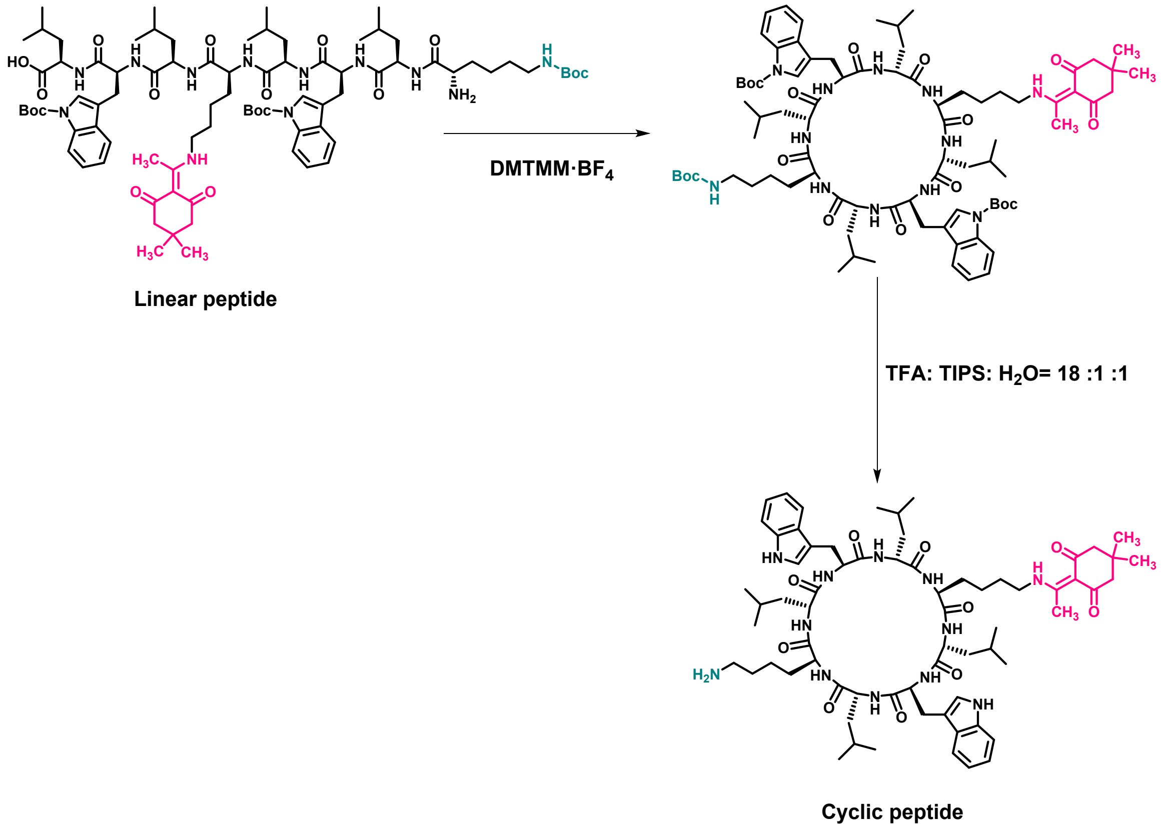

Scheme S2 Example of synthetic process of the cyclic peptide under the cyclization with DMTMM·BF_4 and Boc deprotection using TFA/TIPS/H_2O.

The cyclisation of linear peptide was carried out using 1.2 equivalents of DMTMM·BF_4 in DMF for 72 hours. Specifically, linear peptide (504.4 mg, 0.323 mmol) was dissolved in DMF (100 mL) and DMTMM·BF_4 (127.1 mg, 0.387 mmol) in DMF (10 mL) separately via sonication. The solutions were mixed into a 250 mL round bottle flask and then stirred continuously for 72 h at room temperature. Subsequently, the solution was concentrated by rotary evaporation (50°C) to ~5 mL and then precipitated with cold methanol/water = 1/1 (linear peptide has a better solubility in MeOH). The obtained solid was washed twice by cold methanol/water = 1/1 and dried in a vacuum oven (40 °C, 24 hours).

Cyclo(-L-Lys(Boc)-D-Leu-L-Trp(Boc)-D-Leu-L-Lys(Dde)-D-Leu-L-Trp(Boc)-D-Leu)

Yield: 71% (360.6 mg)

ESI-MS m/z: 1567.7 $[M+Na]^+$ (calculated: 1567.9), 1579.8 $[M+Cl]^-$ (calculated: 1579.9).

^{1}H NMR (400 MHz, Trifluoroacetic acid-*d*) δ(ppm): 8.18-8.03 (d, 2H, Trp), 7.77-7.14 (m, 8H, Trp), 5.33-5.09 (m, 2H, H_αTrp), 4.92-4.51 (m, 6H, H_αLeu and H_αLys), 3.78-3.50 (m, 2H, CH_2-Dde), 3.39-2.95 (m, 10H, CH_2 Trp, C**H_2**-NHBoc Lys and CO-C**H_2**-$C(CH_3)_2$), 2.04-1.21 (m, 51H), 1.21-0.96 (m, 6H, $C(CH_3)_2$ Dde), 1.09-0.62 (m, 24H, CH_3 Leu).

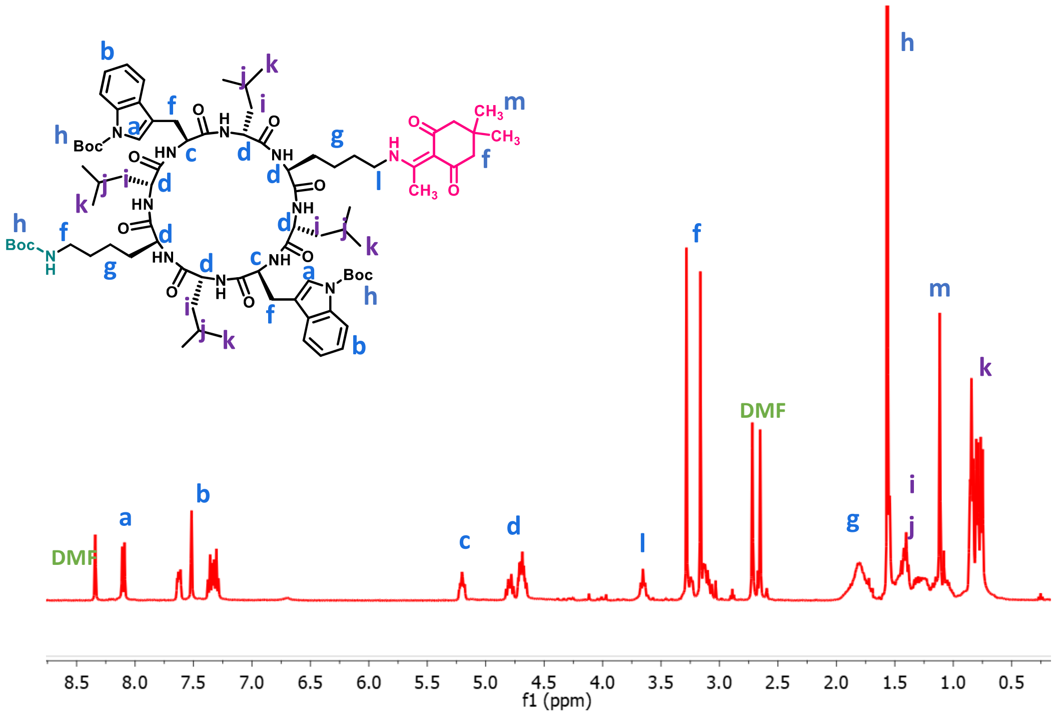

Figure S2 ^{1}H NMR spectrum of protected cyclic peptide in *d*-TFA.

Boc Deprotection

The protecting groups removal of protected cyclic peptides was done by adding a mixture solution of trifluoroacetic acid (TFA, 1.8 mL), triisopropylsilane (TIPS, 0.1 mL) and water (0.1 mL) to protected peptides (150 mg) and stirring for 3 h. The resulting solution was precipitated and then washed twice by cold diethyl ether. The precipitated off-white solid was the deprotected cyclic peptide and then dried in a vacuum oven (40 °C, 3 hours).

Cyclo(-L-Lys-D-Leu-L-Trp-D-Leu-L-Lys(Dde)-D-Leu-L-Trp-D-Leu)

Yield: 78.8% (118.2 mg)

ESI-MS m/z: 1245.7 $[M+H]^+$ (calculated: 1245.7), 1267.6 $[M+Na]^+$ (calculated: 1267.7); 1243.6 $[M-H]^-$ (calculated: 1243.7), 1279.6 $[M+Cl]^-$ (calculated: 1279.7)

^{1}H NMR (400 MHz, Trifluoroacetic acid-*d*) δ(ppm): 7.80-6.89 (m, 8H, Trp), 5.34-5.01 (m, 2H, H_{α}Trp), 4.83-4.53 (m, 6H, H_{α}Leu and H_{α}Lys), 3.81-3.50 (m, 2H, CH_2-Dde), 3.43-2.98 (m, 10H, CH_2 Trp, **CH_2**-NHBoc Lys and CO-**CH_2**-$C(CH_3)_2$), 2.09-1.07 (m, 30H), 1.07-0.51 (m, 24H, CH_3 Leu).

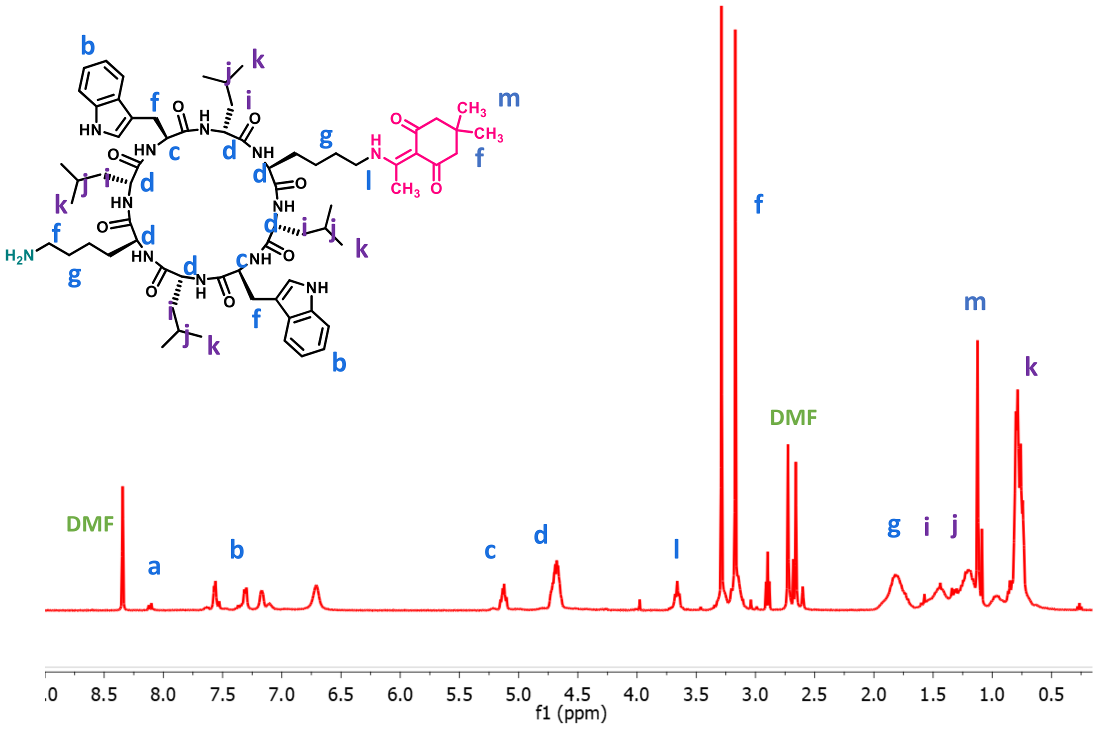

Figure S3 ^{1}H NMR spectrum of cyclic peptide in *d*-TFA.

Synthesis of DMTMM·BF_4 coupling agent

2-Chloro-4,6-dimethoxy-1,3,5-triazine (3.6959 g, 21.05 mmol) was suspended in water (55 mL) and then N-methylmorpholine (2.5 mL, 22.8 mmol) was added in the same bottle. Under agitation for 20 minutes, the solid was entirely dissolved and showed a colourless solution. Sodium tetrafluoroborate (2.7853 g, 25.37 mmol) was dissolved in water (18.5 mL) in another vial to make the stocking solution, which was added dropwise into the reaction mixture over 5 minutes. Crystallisation began straightway. The resulting mixture was stirred for a further 80 minutes and then the product was collected by vacuum filtration. The solid was washed with water (2 × 40 mL), followed by methanol wash (55 mL). After drying in a vacuum oven overnight, white powder was obtained.

Yield: 4.857 mg (71.5%)

^{1}H NMR (400 MHz, MeCN-d_3, ppm): δ= 4.52-4.40 (d, 2H, N-C$\mathbf{H_2}$-CH_2-O), 4.13 (s, 6H, C$\mathbf{H_3}$-O), 4.08-3.92 (m, 2H, N-C$\mathbf{H_2}$-CH_2-O), 3.84-3.62 (m, 4H, N-CH_2-C$\mathbf{H_2}$-O), 3.40 (s, 3H, C$\mathbf{H_3}$-N).

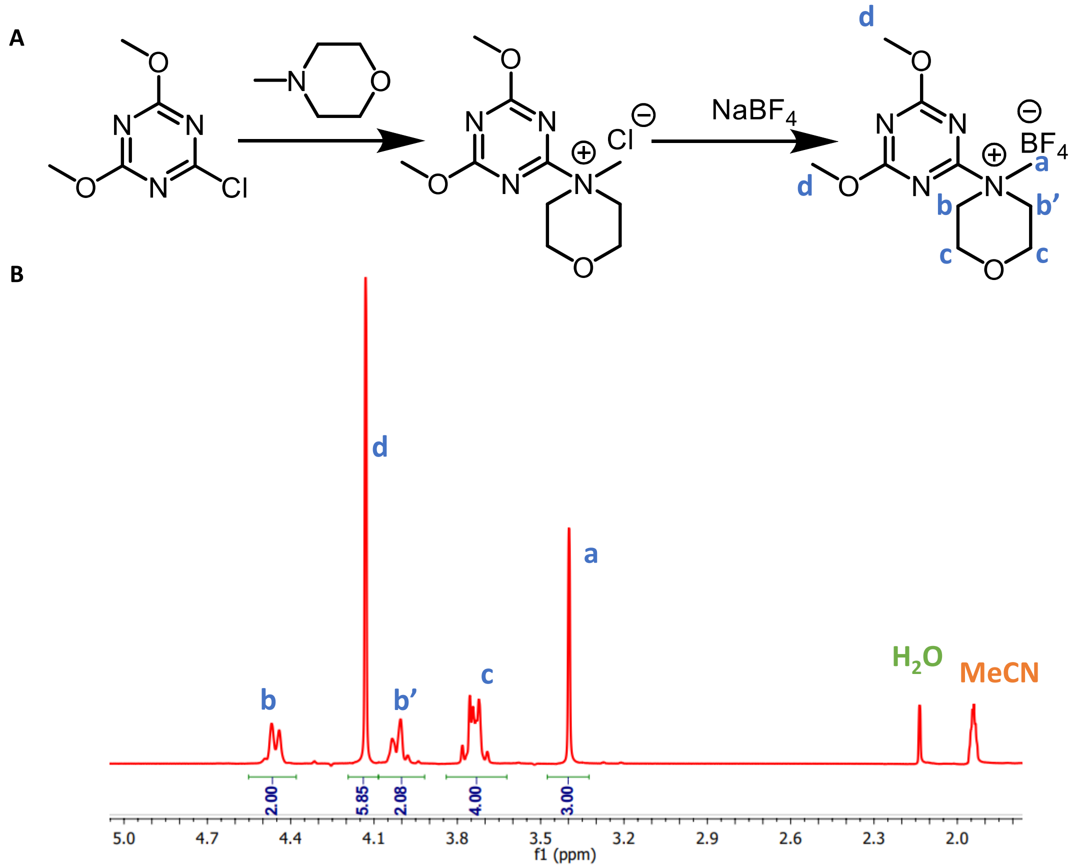

Figure S4 A. Synthetic route to DMTMM·BF_4. B. ^1H NMR of DMTMM·BF_4 in MeCN-d_3.

Polymer synthesis

RAFT agents

(Propanoic acid)yl butyl trithiocarbonate (PABTC) and (4-cyanopentanoic acid)yl ethyl trithiocarbonate (CPAETC) were provided by our group.

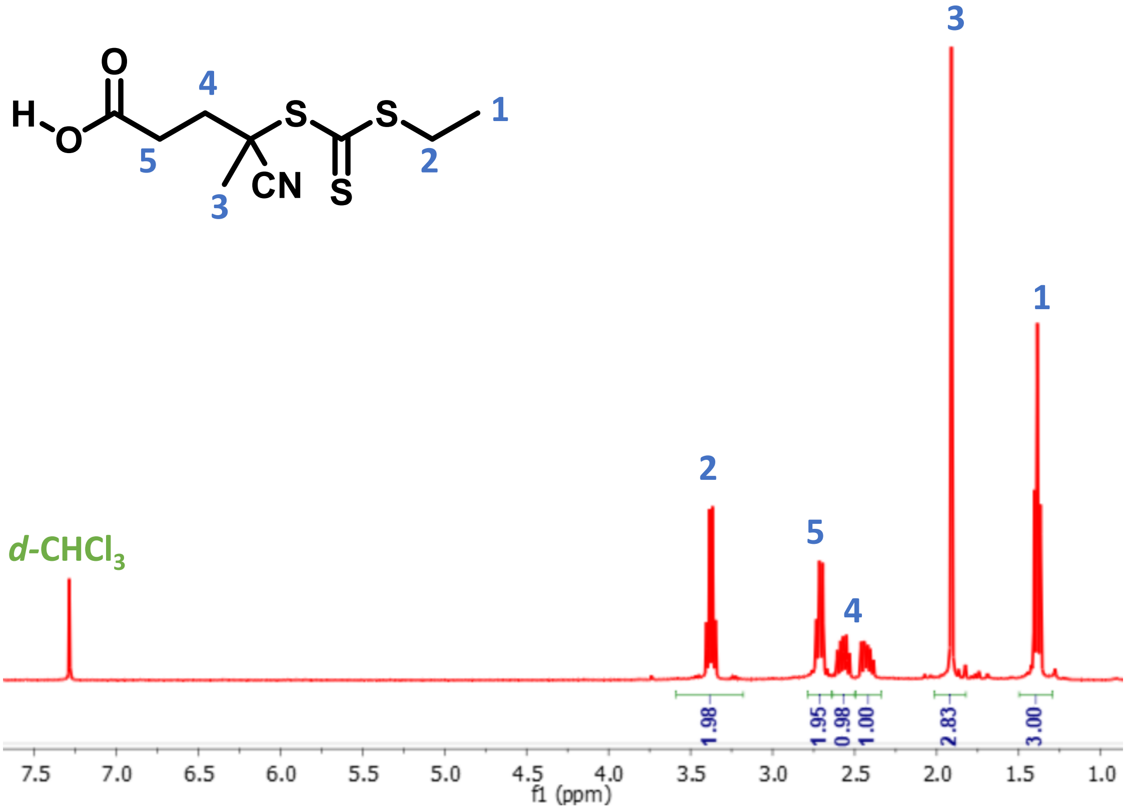

Figure S5 ^{1}H NMR spectrum of CPAETC in *d*-$CHCl_3$.

^{1}**H NMR** (400MHz, *d*-chloroform, ppm): δ= 3.48-3.24 (q, 2H, S-C$\mathbf{H_2}$-CH_3), 2.84-2.60 (m, 2H, C(O)-C$\mathbf{H_2}$-CH_2-), 2.60-2.22 (m, 2H, C(O)-CH_2-C$\mathbf{H_2}$-), 1.95-1.80 (s, 3H, C(CN)(C$\mathbf{H_3}$)), 1.46-1.25 (t, 3H, S-CH_2-C$\mathbf{H_3}$).

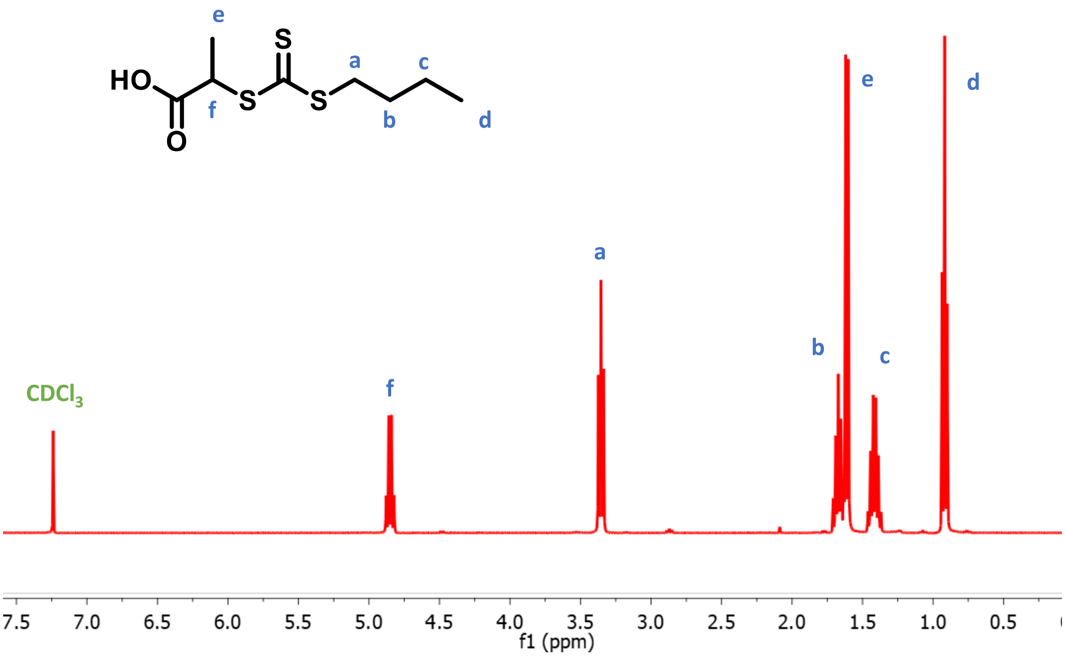

Figure S6 ^{1}H NMR spectrum of PABTC in *d*-$CHCl_3$.

^{1}H NMR (400MHz, *d*-chloroform, ppm): δ= 4.93-4.77 (q, 1H, CO-C**H**(CH_3)), 3.44-3.27 (t, 2H, S-C$\mathbf{H_2}$-CH_3), 1.78-1.56 (m, 5H, S-CH_2-C$\mathbf{H_2}$-CH_2-, CO-CH(C$\mathbf{H_3}$)), 1.51-1.31 (m, 2H, S-CH_2-CH_2-C$\mathbf{H_2}$-), 0.98-0.85 (t, 3H, -CH_2-C$\mathbf{H_3}$).

RAFT polymerization of DEAEMA

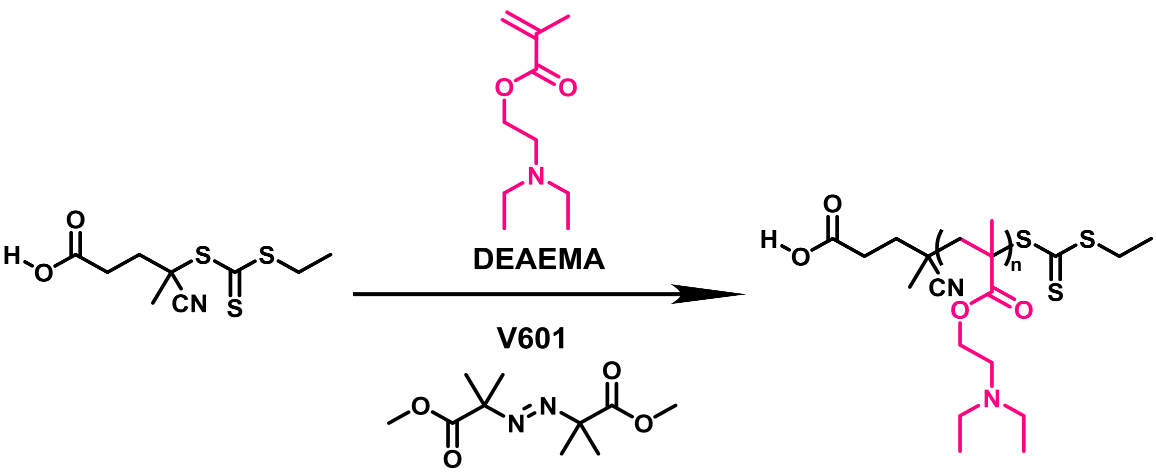

Scheme S3 Preparation of homopolymer of 2-(diethylamino)ethyl methacrylate (DEAEMA).

DP$_{24}$: The mixture solution included the monomer DEAEMA (1001.8 mg, 5.40 mmol), CTA (56.9 mg, 0.22 mmol), initiator V601 20 mg/mL stock solution (124.4 µL, 0.011 mmol) and solvent dioxane (1.49 mL). The vial was equipped with a magnetic stirrer and bubbled with N_2 at room temperature for 15 min, then put into an oil bath set at 70°C and stirred constantly for 15 hours. After polymerization, the vial was removed from the oil bath. When it was cooled down to room temperature, the vial was opened to the air. The excess solvent and unreacted monomers were removed by an evaporator. The final product was a yellow viscous liquid. The conversion of polymerization was monitored by ^{1}H NMR (96%).

DP$_{12}$: The mixture solution included the monomer DEAEMA (2000.3 mg, 10.80 mmol), CTA (218.6 mg, 0.83 mmol), initiator V601 20 mg/mL stock solution (248.6 µL, 0.022 mmol) and solvent dioxane (2.98 mL). The conversion was monitored by ^{1}H NMR (91%).

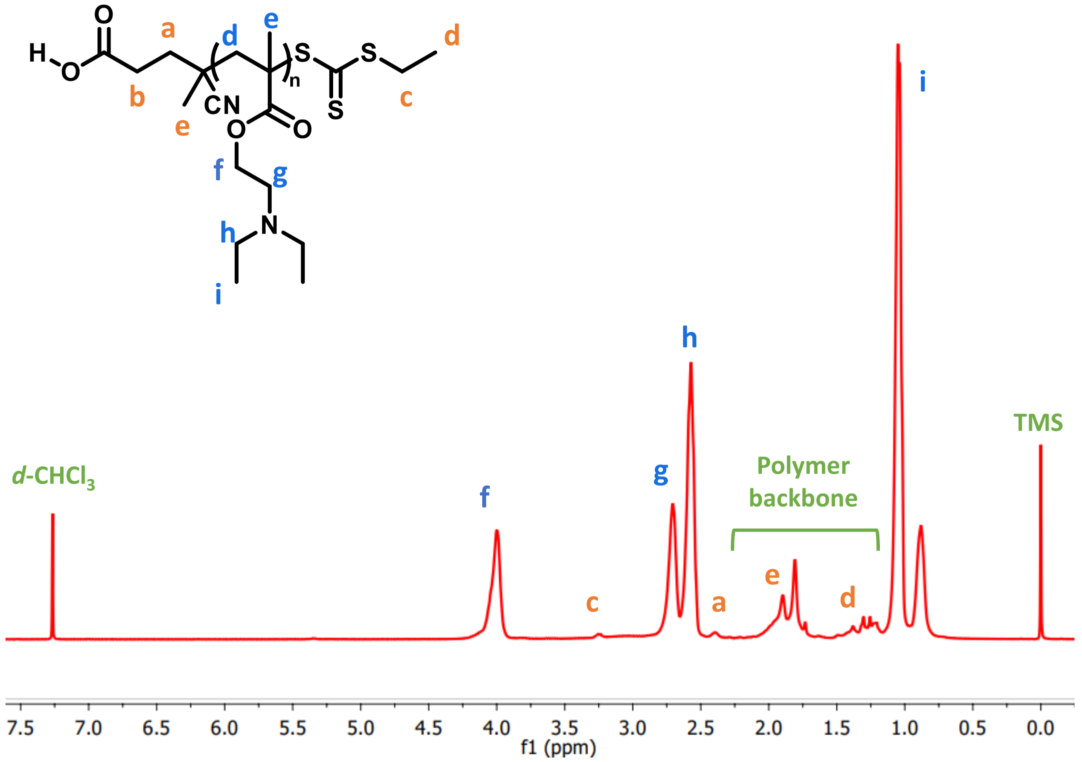

Figure S7 ^{1}H NMR spectrum of pDEAEMA$_{24}$ in *d*-$CHCl_3$.

^{1}H NMR (400MHz, *d*-chloroform, ppm): δ= 4.20-3.90 (m, 48H, CO-O-C**H$_2$**), 2.85-2.70 (m, 48H, CO-O-CH$_2$-C**H$_2$**), 2.70-2.50 (m, 96H, N-C**H$_2$**-CH$_3$), 2.2-1.2 (m, 126H, protons on the backbone, S-CH$_2$-C**H$_3$**, C(CN)(C**H$_3$**)), 1.20-0.75 (m, 144H, N-CH$_2$-C**H$_3$**).

RAFT polymerization of DMAEMA

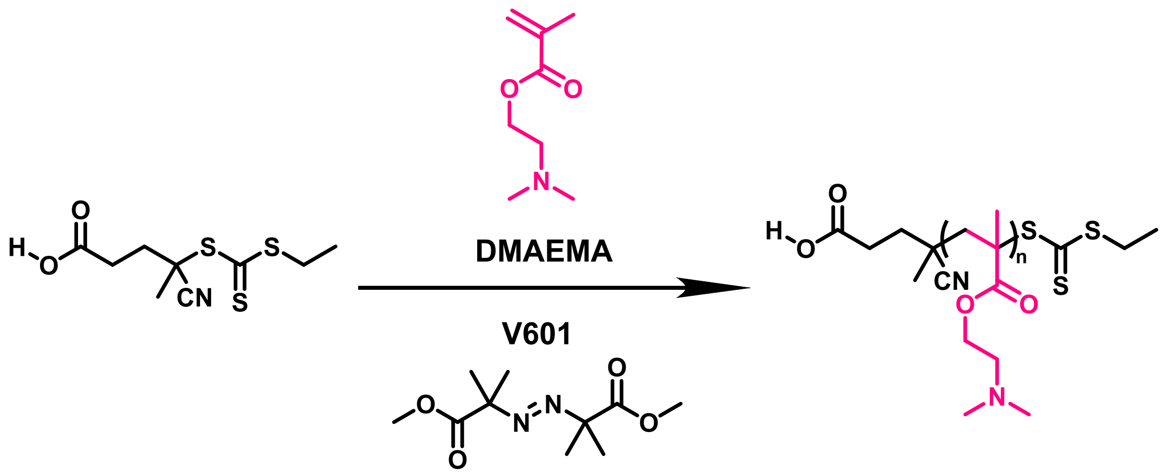

Scheme S4 Preparation of homopolymer of 2-(dimethylamino)ethyl methacrylate (DMAEMA).

DP$_{23}$: The mixture solution included the monomer DMAEMA (2001.4 mg, 12.72 mmol), CTA (133.9 mg, 0.51 mmol), initiator V601 20 mg/mL stock solution (292.9 µL, 0.025 mmol) and solvent dioxane (3.922 mL). The vial was equipped with a magnetic stirrer and sealed with a rubber seal. The solution was deoxygenated through bubbling with N_2 for 25 min, then put into an oil bath set at 70°C and stirred constantly for 21 hours. After polymerization, the vial was removed from the oil bath. When it was cooled down to room temperature, the vial was opened to the air. The purification was achieved by precipitating solution mixture into cold hexane twice and dried in a vacuum oven overnight. The final product was a yellow solid. The conversion of polymerisation was monitored by ^{1}H NMR (94%). (Yield: 95.1%, 2029.9 mg)

DP$_{11}$: The mixture solution included the monomer DEAEMA (2000.8 mg, 12.72 mmol), CTA (257.8 mg, 0.978 mmol), initiator V601 20 mg/mL stock solution (292.9 µL, 0.025 mmol) and solvent dioxane (3.922 mL). The conversion was monitored by ^{1}H NMR (87%). (Yield: 85.0%, 1919.6 mg)

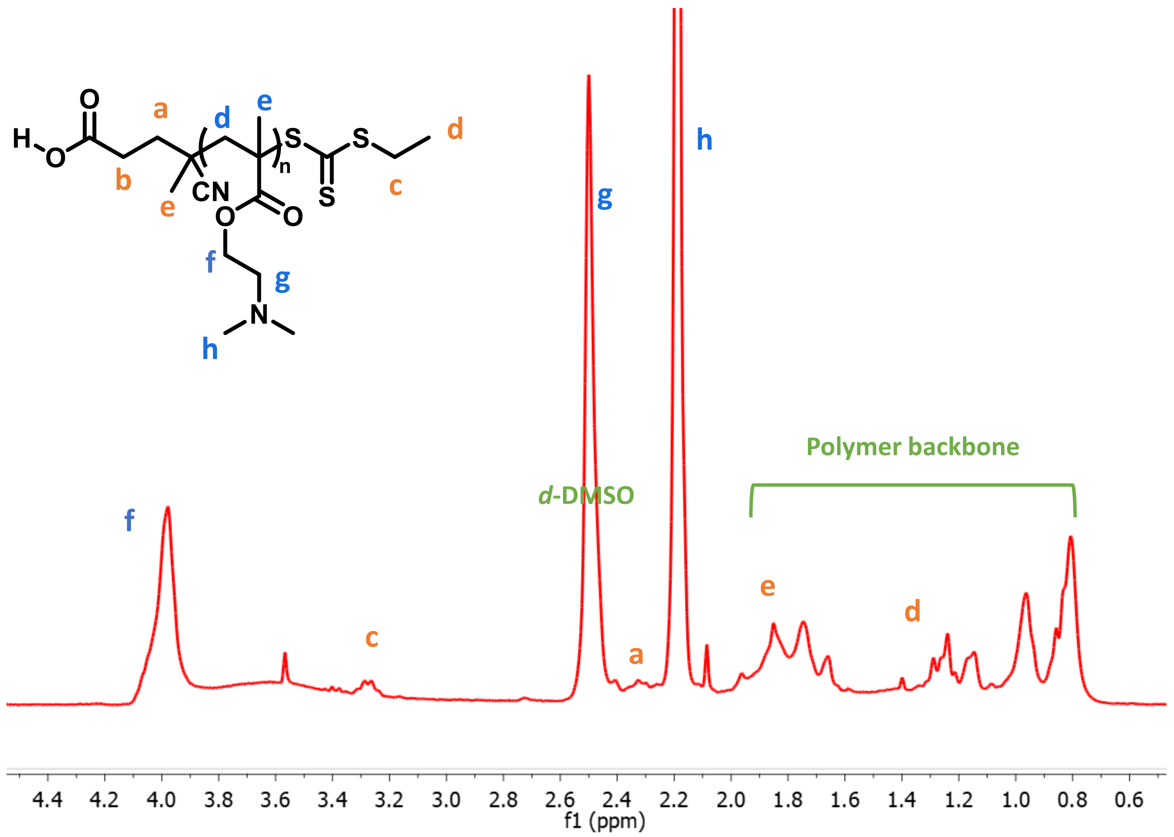

Figure S8 ^{1}H NMR spectrum of pDMAEMA$_{11}$ in *d*-DMSO.

^{1}H NMR (400MHz, *d*-DMSO, ppm): δ= 4.14-3.85 (m, 22H, CO-O-C**H$_2$**), 2.60-2.42 (m, 22H, CO-O-CH$_2$-C**H$_2$**), 2.26-2.11 (m, 66H, N-(C**H$_3$**)$_2$), 2.0-0.7 (m, 61H, protons on the backbone, S-CH$_2$-C**H$_3$**, C(CN)(C**H$_3$**)).

End group removal of RAFT polymers

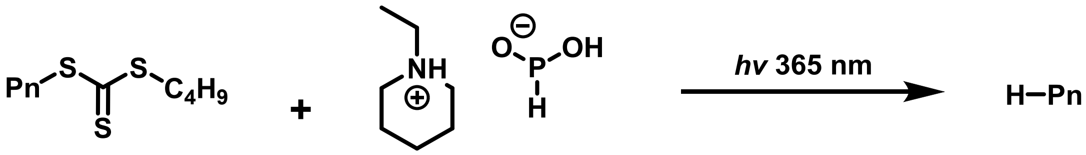

Scheme S5 The mechanism of end group removal using the photoinduced method with EPHP.

General protocol

$pDEAEMA_{24}$ (780 mg, 165.6 µmol) and *N*-ethylpiperidine hypophosphite (EPHP, 445.2 mg, 2.48 mmol, 15 molar equiv.) were dissolved in 4 mL DMF with a magnetic stirrer in a 7 mL vial. Argon was bubbled through the mixture solution for 20 min with stirring. The reaction vessel was positioned under the UV light source for 24 hours and end group removal was initiated upon irradiation. The resulting polymer ($pDEAEMA_{24}$-H) was purified by dialysis (Spectra/Por 3 Dialysis Membranes (1000 MWCO)) in MeOH/DI water system for 2 days followed by lyophilization.

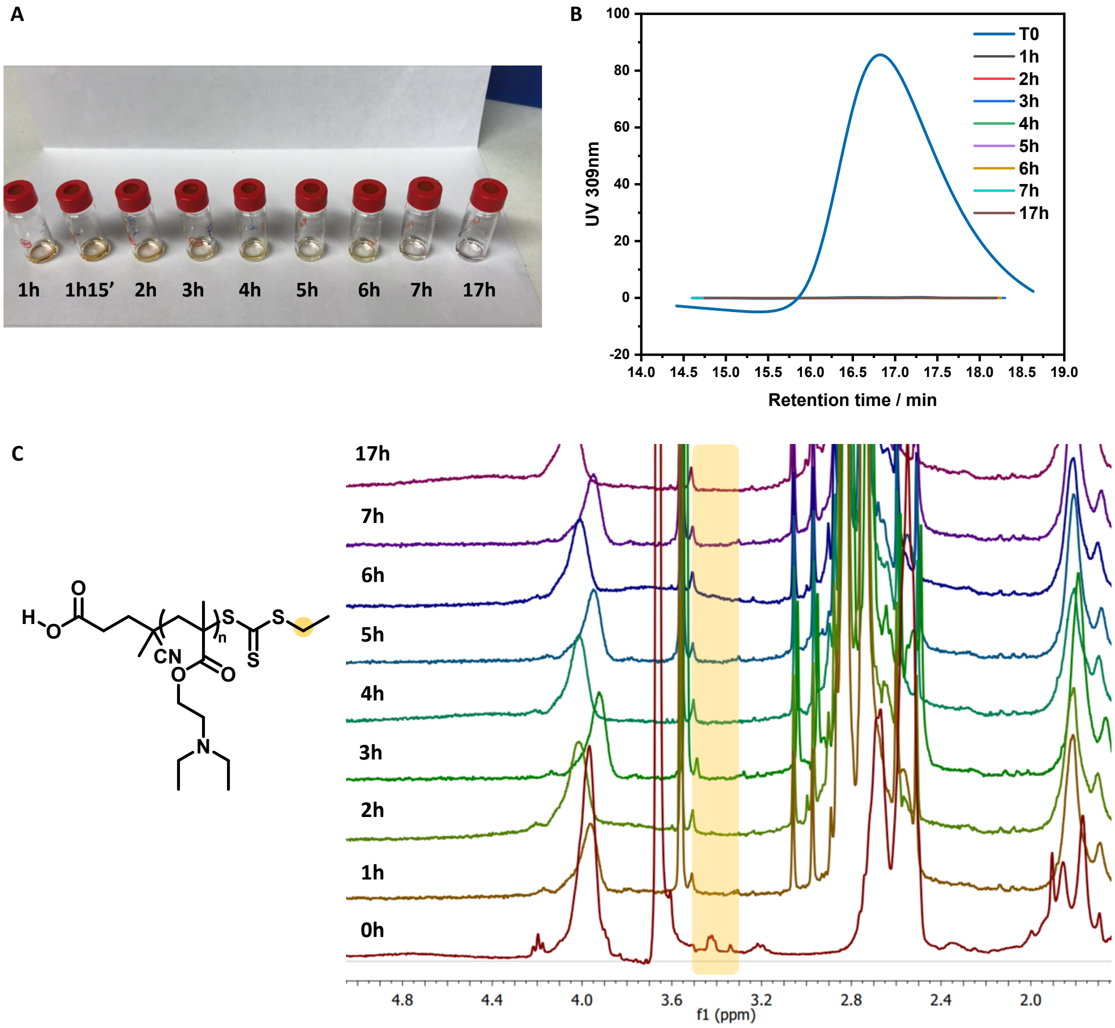

Figure S9 Kinetic study of the photo-induced end group removal of pDEAMEA$_{24}$. The comparison of the colour evolution (**A**), the UV absorption (**B**) and NMR spectra (**C**) under different time points.

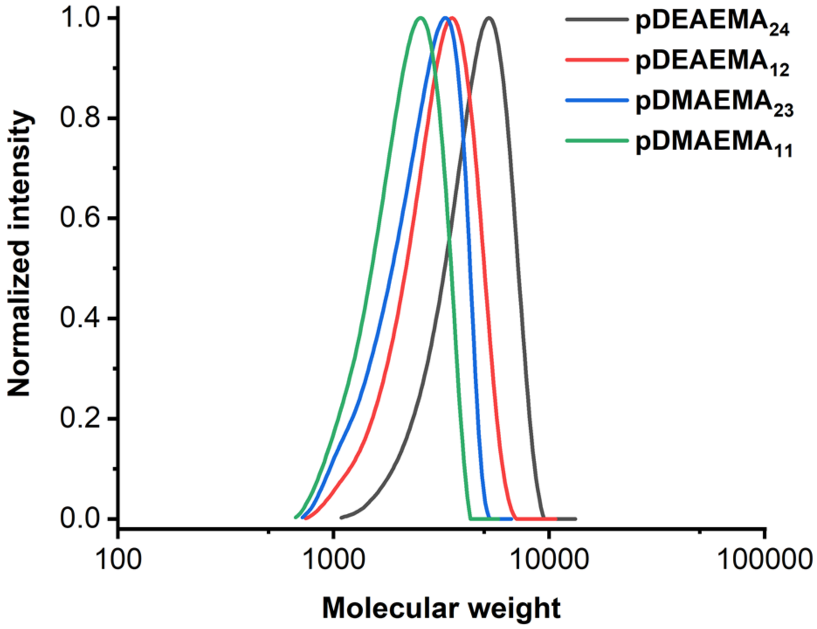

Figure S10 Normalized GPC RI molecular weight distributions for $pDEAEMA_{12}$, $pDEAEMA_{24}$, $pDMAEMA_{23}$ and $pDMAEMA_{11}$ in THF after end group removal.

Table S1 Summary of characterisation details of synthetic RAFT polymers in GPC(THF).

**polymer**	**DP** ^{A}	**Conv.** ^{A} **(%)**	$M_{n,th}$ ^{A} **(g/mol)**	$M_{n,SEC}$ ^{B} **(g/mol)**	$Đ$ ^{B}
$pDEAEMA_{24}$	24	96	4,700	4,100	1.14
$pDEAEMA_{12}$	12	91	2,500	2,800	1.16
$pDMAEMA_{23}$	23	94	3,900	2,300	1.16
$pDMAEMA_{11}$	11	87	2,000	1,900	1.14

[A] Monomer conversion was obtained by the comparison between the proton on the polymer and the proton on the monomer in the crude solution from ^1H NMR spectroscopy. Degree of polymerisation and theoretical molecular weight were calculated from the conversion.

[B] Determined by SEC analysis in THF with DRI and UV 309 nm detectors and PMMA calibration.

Cyclic peptide-polymer conjugate synthesis

General protocol

Synthesis of DdeNH-CP-PEG5k

20.0 mg of Dde-CP-NH_2 (1 equiv., 16.1 μmol) was dissolved in 1 mL DMF (extra dry). In another vial, 121.5 mg of PEG5k (1.5 equiv., 24.1 μmol), 9.16 mg of HATU (1.5 equiv., 22.5 μmol) and 4.9 mg of NMM (3 equiv., 48.2 μmol) were entirely dissolved in 1 mL DMF (extra dry) for 30 mins. The activated polymer solution was dropped into the cyclic peptide solution, and the mixture was reacted at room temperature overnight. The purification was achieved by two steps: first, the mixture solution was precipitated in cold diethyl ether; Then the obtained solid was redissolved in 1.5 mL DCM and added dropwise into 9 mL diethyl ether to get the white precipitate, which process was repeated twice. Finally, the obtained conjugates were redissolved in pure water and dried in a freezer dryer to yield the white solid. (Yield: 85.6%, 85.6 mg)

Synthesis of NH_2-CP-PEG5k

The Dde protecting group removal of protected cyclic peptides was achieved by dissolving CP-PEG5k conjugates in a 4% hydrazine DMF solution and stirring it for 3 h. The mixture solution was precipitated twice in cold diethyl ether to obtain solid product. The obtained conjugates were redissolved in pure water and dried in a freezer dryer to yield the white solid. GPC (DMF/LiBr): M_n = 13,100 g mol^{-1}, $Đ$ = 1.17. (Yield: 73%, 55.2 mg)

Synthesis of $pDEAEMA_{24}$-CP-PEG5k

20.2 mg of NH_2-CP-PEG5k (1 equiv., 3.29 μmol) was dissolved in 0.5 mL DMF (extra dry). In another vial, 45 mg of $pDEAEMA_{24}$ (3 equiv., 9.89 μmol), 3.76 mg of HATU (3 equiv., 9.89 μmol) and 1.5 mg of NMM (4.5 equiv., 14.8 μmol) were entirely dissolved in 1.0 mL DMF (extra dry) and stirred for 20 mins. The activated polymer solution was dropped into the cyclic peptide solution, and the mixture was reacted at room temperature for one day. Then, 3 eq. NMM and 2 eq. HATU were added to the mixture three times to improve the reaction conversion. The purification of amphiphilic conjugates was achieved by precipitation twice using cold diethyl ether. The obtained product was dissolved in pure water and dried in a freezer dryer to yield the white solid. GPC (DMF/LiBr): M_n = 17,900 g mol^{-1}, $Đ$ = 1.15. (Yield: 92%, 32.8 mg)

^{1}H NMR (400MHz, *d*-DMSO, ppm): δ= 10.76 (s, 2H, N**H** Trp), 8.41-7.91 (m, 8H, CO-N**H** on the peptide ring), 7.91-7.55 (m, 3H, CO-N**H** on the polymer arms), 7.55-6.84 (m, 8H, Trp), 4.80-4.53 (m, 2H, 2H, Hα Trp), 4.53-4.26 (m, 6H, Hα Leu and Hα Lys), 4.25-3.93 (m, 48H, CO-O-C$\mathbf{H_2}$), 3.93-3.03 (m, 456H, -C$\mathbf{H_2}$-C$\mathbf{H_2}$-O- PEG backbone), 2.12-1.2 (m, 123H, protons on the backbone, C(CN)(C$\mathbf{H_3}$)), 1.20-0.75 (m, 168H, CH3 Leu, N-CH_2-C$\mathbf{H_3}$).

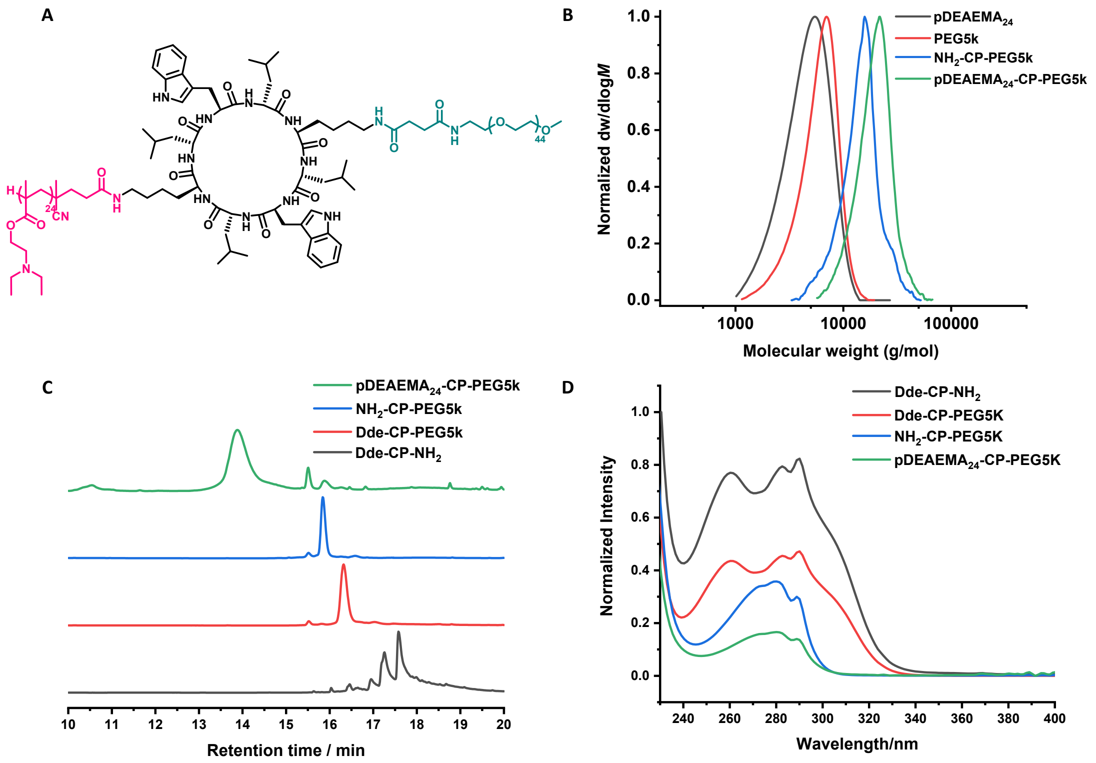

Figure S11 A. Chemical structure of the pDEAEMA$_{24}$-CP-PEG5k conjugate. **B.** Normalized GPC RI molecular weight distributions for PEG5k, pDEAEMA$_{24}$, NH_2-CP-PEG5k and pDEAEMA$_{24}$-CP-PEG5k in DMF + 0.1% LiBr. **C.** HPLC spectra of Dde-CP-NH_2, Dde-CP-PEG5k, NH_2-CP-PEG5k and pDEAEMA$_{24}$-CP-PEG5k under UV 280 nm. **D.** The comparison of the UV absorption of each compound detected by HPLC.

Table S2 GPC data for each precursor for pDEAEMA$_{24}$-CP-PEG5k in DMF + 0.1% LiBr.

Sample	M_n (g mol^{-1})	*Đ*
pDEAEMA$_{24}$	4,000	1.25
PEG5k	5,200	1.19
NH_2-CP-PEG5k	13,100	1.17
pDEAEMA$_{24}$-CP-PEG5k	17,900	1.15

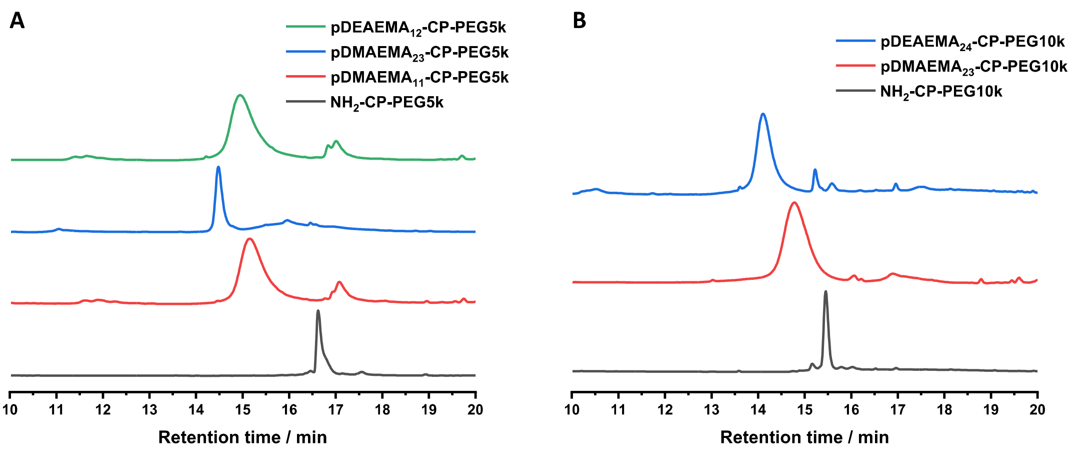

Figure S12 A. HPLC spectra of NH_2-CP-PEG5k, pDMAEMA$_{11}$-CP-PEG5k, pDMAEMA$_{23}$-CP-PEG5k and pDEAEMA$_{12}$-CP-PEG5k under UV 280 nm. **B.** HPLC spectra of NH_2-CP-PEG10k, pDMAEMA$_{23}$-CP-PEG10k and pDEAEMA$_{24}$-CP-PEG10k under UV 280 nm.

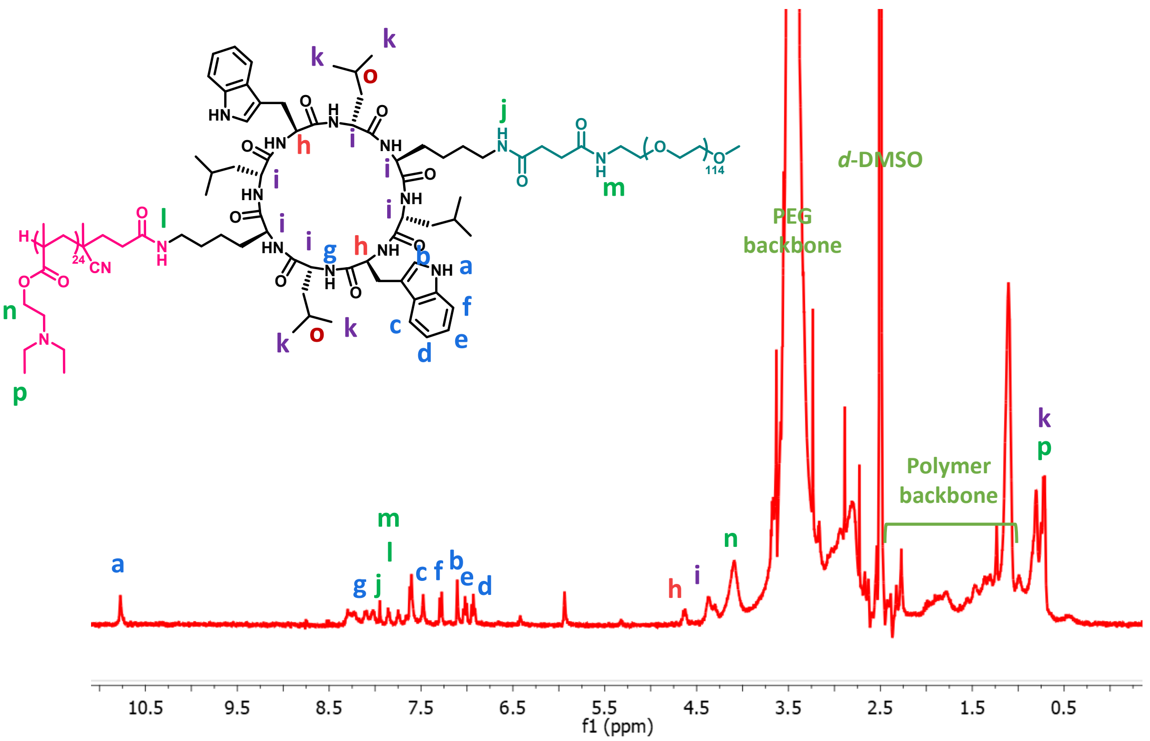

Figure S13 ^{1}H NMR spectrum (400MHz) of pDEAEMA$_{24}$-CP-PEG5k in *d*-DMSO.

General control copolymer synthesis

20.1 mg of linear NH_2-PEG10k (1.0 equiv., 1.98 μmol) was dissolved in 0.5 mL DMF (extra dry). In another vial, 13.2 mg of $pDMAEMA_{23}$ (1.5 equiv., 2.98 μmol), 3.76 mg of HATU (1.13 equiv., 2.98 μmol) and 0.60 mg of NMM (3 equiv., 5.964 μmol) were entirely dissolved in 0.5 mL DMF (extra dry) and stirred for 20 mins and then mixed with the PEG solution and left overnight. The purification was achieved by precipitation twice using cold diethyl ether. The obtained product was dissolved in pure water and dried in a freezer dryer to yield the white solid. GPC (THF): M_n = 17,400 g mol^{-1}, $Đ$ = 1.14. (Yield: 31%, 8.7 mg)

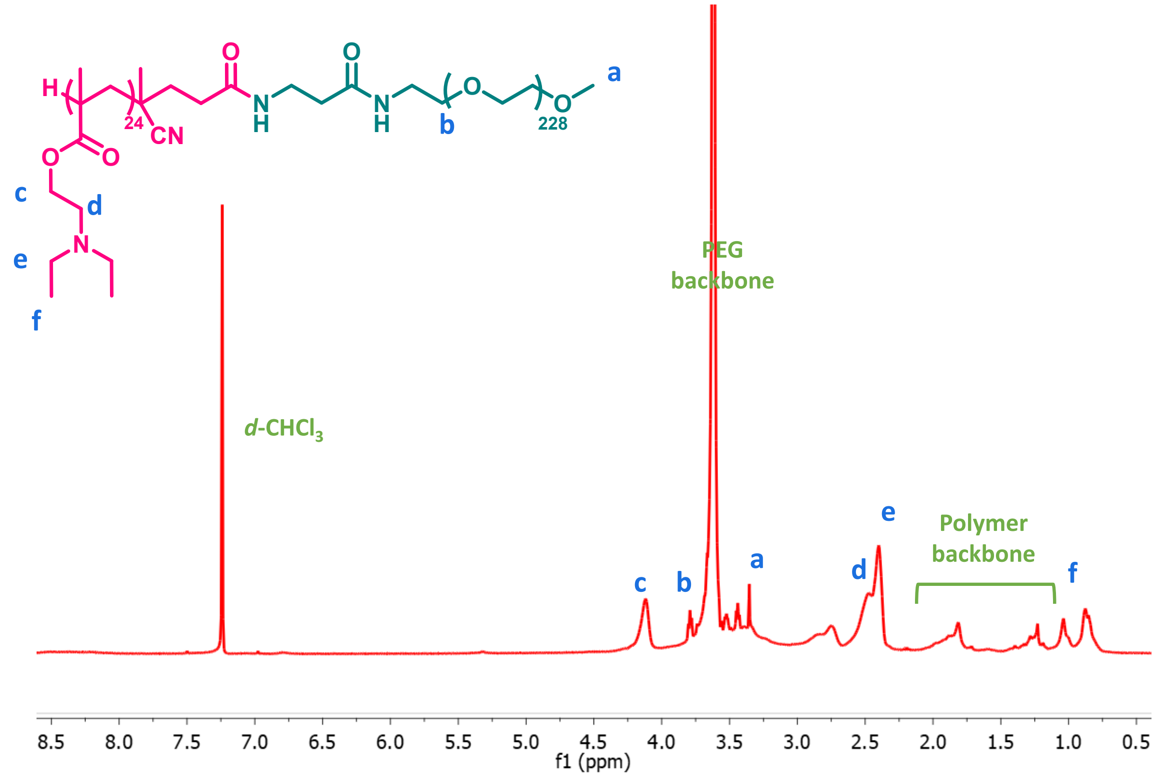

Figure S14 ^{1}H NMR spectrum (400MHz) of $pDMAEMA_{23}$-PEG10k in *d*-$CHCl_3$.

^{1}H NMR (400MHz, *d*-$CHCl_3$, ppm): δ= 4.32-3.98 (m, 48H, CO-O-C**H₂**), 3.89-3.75 (t, 2H, NH-CH_2-C**H_2**-O-), 3.75-3.40 (m, 912H, -C**H_2**-C**H_2**-O- PEG backbone), 3.35 (s, 3H, -O-C**H_3**), 2.67-2.27 (m, 144H, N-C**H_2**-CH_3, N-C**H_2**-CH_2-), 2.12-1.2 (m, 123H, protons on the backbone, C(CN)(C**H_3**)), 1.20-0.75 (m, 144H, N-CH_2-C**H_3**).

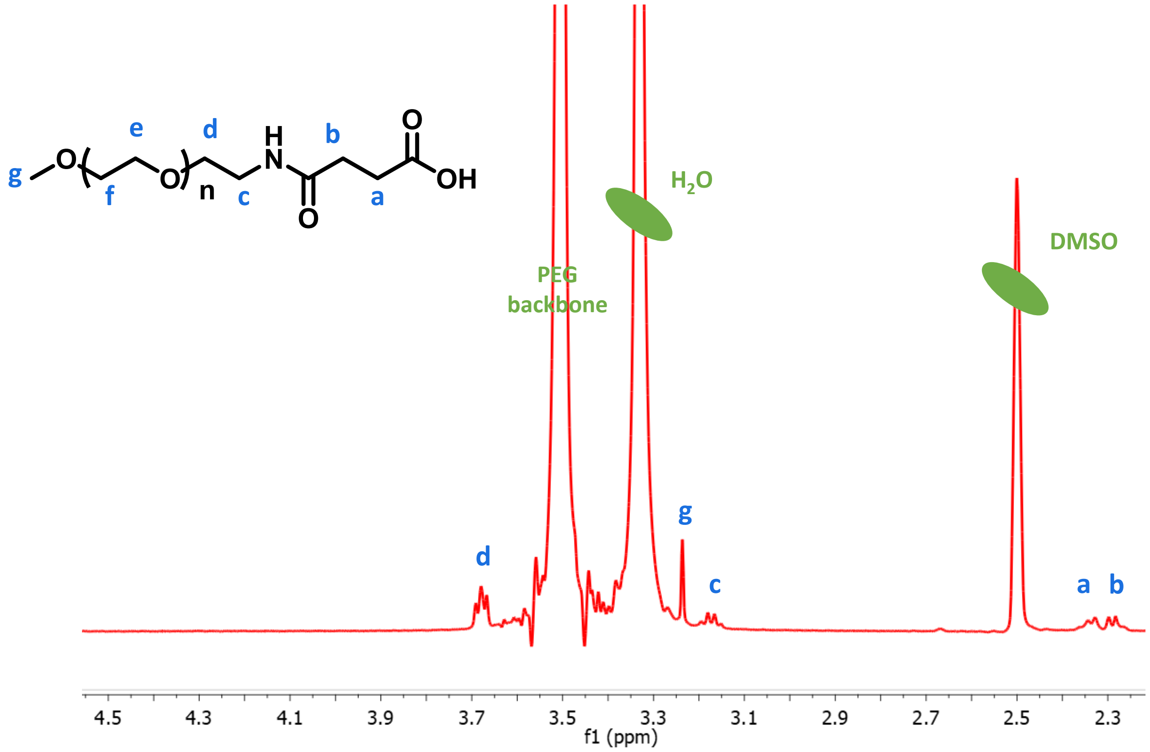

Figure S15 ^{1}H NMR spectrum (400MHz) of linear PEG10k in *d*-DMSO.

^{1}H NMR (400MHz, *d*-$CHCl_3$, ppm): δ= 3.75-3.65 (t, 3H), 3.6-3.4 (m, 912H), 3.25 (s, 3H), 3.20-3.15 (m, 2H), 2.4-2.25 (m, 4H).

SANS fitting data summary

1. Static measurement

1.1 One-arm CP-PEG conjugates

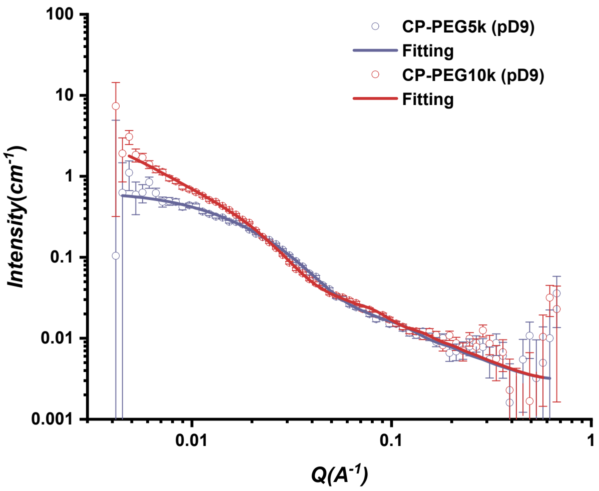

Figure S16 Scattering profiles and respective fits of CP-PEG5k and CP-PEG10k at pD9. The fitting model was a core-shell cylinder combined with a Gaussian coil.

Table S3 Fitting parameters for CP-PEG5k and CP-PEG10k, using the **Poly_Gauss_Coil** + **Core_Shell_Cylinder** combined model.

Parameter	Fit	pD9	
		CP-PEG5K	CP-PEG10K
Scale		1.0	1.0
Background (1/cm)		0.003	0.003
A_scale		1.0	1.0
A_i_zero (1/cm)	*	0.46174	4.316
A_rg (Å)	*	146.87	485.44
A_polydispersity		1.19	1.19

**B_scale**	*	0.9605	2.3352
**B_sld_core (10^{-6}/ Å^2)**		1.5577	1.5577
**B_sld_shell (10^{-6}/ Å^2)**	*	6.4132	6.4194
**B_sld_solvent (10^{-6}/ Å^2)**		6.42836	6.42836
**B_radius (Å)**		5.0	5.0
**B_thickness (Å)**	*	62.211	97.46
**B_length (Å)**	*	72.198	97.089
**Reduced χ^2**		0.90	1.11

1.2 Control diblock copolymer

Table S4 Fitting parameters for pDMAEMA$_{23}$-PEG5k, using the **Poly_Gauss_Coil + Power_Law** combined model.

Parameter	Fit	pD13
		**pDMAEMA$_{23}$-PEG5k**
**Scale**		1.0
**Background (1/cm)**		0.001
**A_scale**		1.0
**A_i_zero (1/cm)**	*	0.036638
**A_rg (Å)**	*	38.57
**A_polydispersity**		2.33
**B_scale**	*	0.0032107
**B_power**	*	0.53653
**Reduced χ^2**		0.78

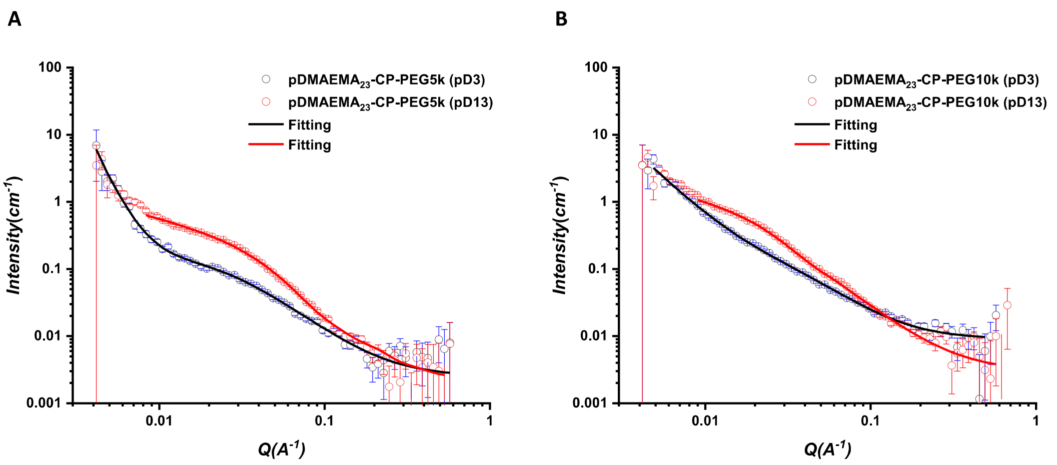

Figure S17 Scattering profiles and respective fits of asymmetric CPPC2 (A) and CPPC3 (B) in pD3 and pD13. The fitting model for pD3 was a Gaussian coil combined with a power law (red line) and for pD13 was a core-shell cylinder model combined with a Gaussian coil (black line).

1.3 pDMAEMA asymmetric conjugates

Table S5 Fitting parameters for pDMAEMA$_{11}$-CP-PEG5k (CPPC1), pDMAEMA$_{23}$-CP-PEG5k (CPPC2), pDMAEMA$_{23}$-CP-PEG10k (CPPC3), using the **Poly_Gauss_Coil + Power_Law** combined model.

Parameter	Fit	pD3	pD3	pD9	pD10	pD11	pD3
		CPPC 1	CPPC 2				CPPC 3
Scale		1.0	1.0	1.0	1.0	1.0	1.0
Background (1/cm)		0.0035	0.0025	0.0035	0.005	0.004	0.009
A_scale		1.0	1.0	1.0	1.0	1.0	1.0
A_i_zero (1/cm)	*	0.12727	0.16277	0.14488	0.15898	0.14792	0.11283
A_rg (Å)	*	71.972	67.786	59.136	64.488	59.824	49.819
A_polydispersity		2.31	2.33	2.33	2.33	2.33	2.32
B_scale	*	$2.1362e^{-08}$	$7.811e^{-11}$	$2.64e^{-10}$	$1.56e^{-09}$	$1.72e^{-09}$	$1.967e^{-05}$
B_power	*	3.2945	4.4513	4.2948	3.9639	3.8791	2.2205
Reduced χ^2		0.89	1.06	0.75	0.81	1.18	1.57

Table S6 Fitting parameters for pDMAEMA$_{11}$-CP-PEG5k (CPPC1), pDMAEMA$_{23}$-CP-PEG5k (CPPC2), pDMAEMA$_{23}$-CP-PEG10k (CPPC3), using the **Poly_Gauss_Coil + Core_Shell_Cylinder** combined model.

Parameter	Fit	pD13	pD12	pD13	pD13
		CPPC 1	CPPC 2		CPPC 3
Scale		1.0	1.0	1.0	1.0
Background (1/cm)		0.007	0.002	0.002	0.003
A_scale		1.0	1.0	1.0	1.0
A_i_zero (1/cm)	*	0.18935	0.22541	0.21815	0.57409
A_rg (Å)	*	67.151	59.921	63.155	82.394
A_polydispersity		2.33	2.33	2.33	2.33
B_scale	*	0.011256	$5.3306e^{-05}$	0.01008	0.0027583
B_sld_core (10^{-6}/ Å^2)		1.5577	1.5577	1.5577	1.5577
B_sld_shell (10^{-6}/ Å^2)	*	6.1107	3.1927	5.9819	5.8518
B_sld_solvent (10^{-6}/ Å^2)		6.42836	6.42836	6.42836	6.42836
B_radius (Å)		5.0	5.0	5.0	5.0
B_thickness (Å)	*	52.107	50.385	34.667	75.069
B_length (Å)	*	208.58	419.2	495.18	999.91
Reduced χ^2		1.70	0.83	1.79	2.27

1.4 pDEAEMA asymmetric conjugates

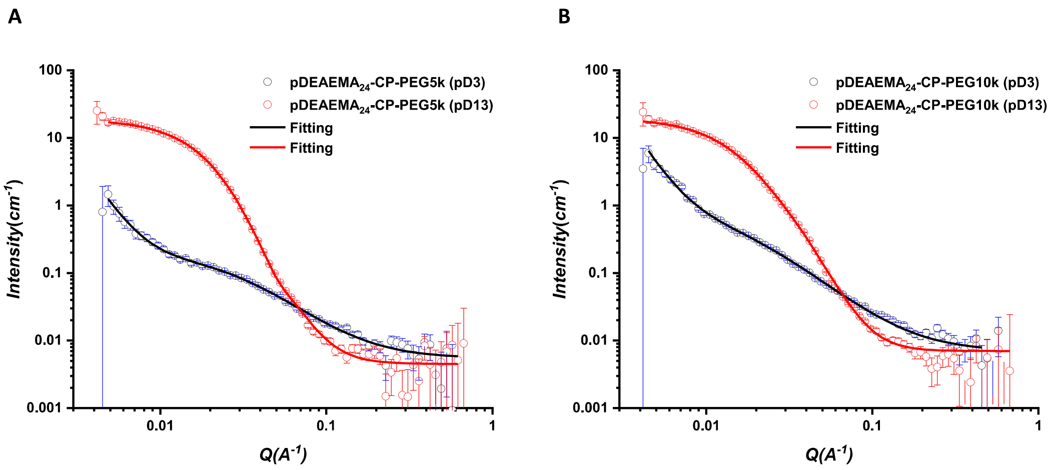

Figure S18 Scattering profiles and respective fits of asymmetric CPPC5 (A) and CPPC6 (B) in pD3 and pD13. The fitting model for pD3 was a Gaussian coil combined with a power law (red line) and for pD13 was a core-shell cylinder model combined with a Gaussian coil (black line).

Table S7 Fitting parameters for pDEAEMA$_{12}$-CP-PEG5k (CPPC4), pDEAEMA$_{24}$-CP-PEG5k (CPPC5), pDEAEMA$_{24}$-CP-PEG10k (CPPC6), using the **Poly_Gauss_Coil + Power_Law** combined model.

Parameter	Fit	pD3		
		CPPC 4	CPPC 5	CPPC 6
Scale		1.0	1.0	1.0
Background (1/cm)		0.007	0.0055	0.007
A_scale		1.0	1.0	1.0
A_i_zero (1/cm)	*	0.54273	0.16753	0.63109
A_rg (Å)	*	84.51	62.01	102.68
A_polydispersity		2.31	2.31	2.32
B_scale	*	8.4123e^{-09}	2.8487e^{-09}	3.4278e^{-08}
B_power	*	3.6532	3.7084	3.4433
Reduced χ^2		1.92	0.79	1.67

Table S8 Fitting parameters for pDEAEMA$_{12}$-CP-PEG5k (CPPC4), pDEAEMA$_{24}$-CP-PEG5k (CPPC5), pDEAEMA$_{24}$-CP-PEG10k (CPPC6), using the **Poly_Gauss_Coil + Core_Shell_Cylinder** combined model.

Parameter	Fit	pD13	pD9	pD10	pD11	pD12	pD13	pD13
		CPPC4			CPPC5			CPPC6
Scale		1.0	1.0	1.0	1.0	1.0	1.0	1.0
Background (1/cm)		0.007	0.0035	0.004	0.004	0.005	0.0045	0.006
A_scale		1.0	1.0	1.0	1.0	1.0	1.0	1.0
A_i_zero (1/cm)	*	0.3091	0.5964	0.3911	0.3348	0.2738	0.0238	$3.06e^{-07}$
A_rg (Å)	*	149.99	120.25	108	124	132.9	137.8	97.411
A_polydispersity		2.31	2.31	2.31	2.31	2.31	2.31	2.31
B_scale	*	0.1109	0.1537	0.2519	0.0616	0.1422	0.0042	0.0710
B_sld_core (10^{-6}/ Å^2)		1.5577	1.5577	1.5577	1.5577	1.5577	1.5577	1.5577
B_sld_shell (10^{-6}/ Å^2)	*	6.0254	6.1851	6.2111	5.9061	6.0292	3.9329	5.8576
B_sld_solvent (10^{-6}/ Å^2)		6.42836	6.42836	6.42836	6.42836	6.42836	6.42836	6.42836
B_radius (Å)		5.0	5.0	5.0	5.0	5.0	5.0	5.0
B_thickness (Å)	*	46.213	73.546	71.208	68.76	69.168	65.362	36.669
B_length (Å)	*	66.416	500.93	259.87	127.68	205.62	105.88	147.98
Polydispersity of thickness[1]		0.3708	0.2116	0.2269	0.2444	0.2590	0.2676	0.5126
Reduced χ^2		2.43	1.29	2.35	1.48	3.24	1.67	6.72

[1] A lognormal function was applied for the calculation involving polydispersity.

2. Time-resolved experiment of CPPC5

General protocol

The addition of GdL was 4 mg/mL. Based on the volume of CPPC5 (pD13), solid GdL was calculated and weighted. Before the measurement, the solid sugar was quickly mixed with the CPPC5 solution and injected into the cuvette, followed by loading it into the instrument.

Table S9 Fitting parameters for pDEAEMA$_{24}$-CP-PEG5k (CPPC5) in first 50 mins, using the **Poly_Gauss_Coil + Core_Shell_Cylinder** combined model.

Parameter	Fit	10 min	20 min	30 min	40 min	50 min
**Scale**		1.0	1.0	1.0	1.0	1.0
**Background (1/cm)**		0.0012	0.002	0.004	0.004	0.004
**A_scale**		1.0	1.0	1.0	1.0	1.0
**A_i_zero (1/cm)**	*	1.8446	0.42486	0.22993	0.40748	0.48158
**A_rg (Å)**	*	174.64	87.177	69.668	108.43	124.32
**A_polydispersity**		2.31	2.31	2.31	2.31	2.31
**B_scale**	*	0.0081882	0.0094918	0.011039	0.31506	0.44098
**B_sld_core (10^{-6}/ Å^2)**		1.5577	1.5577	1.5577	1.5577	1.5577
**B_sld_shell (10^{-6}/ Å^2)**	*	5.7995	5.9082	6.0043	6.3884	6.4026
**B_sld_solvent (10^{-6}/ Å^2)**		6.42836	6.42836	6.42836	6.42836	6.42836
**B_radius (Å)**		5.0	5.0	5.0	5.0	5.0
**B_thickness (Å)**	*	56.407	52.077	52.568	57.109	53.527
**B_length (Å)**	*	> 1000	516.34	316.5	95.244	54.71
**Reduced χ^2**		0.96	1.10	1.28	0.87	0.88

Table S10 Fitting parameters for pDEAEMA$_{24}$-CP-PEG5k (CPPC5) at 60 min, comparing two fitting models including the **Poly_Gauss_Coil** + **Core_Shell_Cylinder** and **Poly_Gauss_Coil** + **Power_Law**.

Core-shell cylinder + Gaussian coil			Gaussian coil + power law		
**Parameter**	**Fit**	**60 min**	**Parameter**	**Fit**	**60 min**
**Scale**		1.0	**Scale**		1.0
**Background (1/cm)**		0.004	**Background (1/cm)**		0.004
**A_scale**		1.0	**A_scale**		1.0
**A_i_zero (1/cm)**	*	0.39021	**A_i_zero (1/cm)**	*	0.49025
**A_rg (Å)**	*	98.155	**A_rg (Å)**	*	106.78
**A_polydispersity**		2.31	**A_polydispersity**		2.31
**B_scale**	*	0.10392	**B_scale**	*	$3.4119e^{-19}$
**B_sld_core (10^{-6}/ Å^2)**		1.5577	**B_power**	*	7.8821
**B_sld_shell (10^{-6}/ Å^2)**	*	6.3725	**Reduced χ^2**		1.18
**B_sld_solvent (10^{-6}/ Å^2)**		6.42836			
**B_radius (Å)**		5.0			
**B_thickness (Å)**	*	56.286			
**B_length (Å)**	*	0.00017183			
**Reduced χ^2**		1.07			

Table S11 Fitting parameters for pDEAEMA$_{24}$-CP-PEG5k (CPPC5) after 1h, using the **Poly_Gauss_Coil + Power_Law** combined model.

Parameter	Fit	70 min	80 min	90 min	100 min	110 min	120 min
**Scale**		1.0	1.0	1.0	1.0	1.0	1.0
**Background (1/cm)**		0.005	0.005	0.005	0.005	0.005	0.005
**A_scale**		1.0	1.0	1.0	1.0	1.0	1.0
**A_i_zero (1/cm)**	*	0.4266	0.3983	0.3799	0.37475	0.35225	0.33842
**A_rg (Å)**	*	101.35	96.671	97.391	98.282	95.653	90.756
**A_polydispersity**		2.31	2.31	2.31	2.31	2.31	2.31
**B_scale**	*	$1.46e^{-13}$	$1.59e^{-16}$	$1.45e^{-11}$	$1.54e^{-11}$	$1.67e^{-08}$	$6.7434e^{-10}$
**B_power**	*	5.4937	6.8847	4.5378	4.5729	3.2505	3.894
**Reduced χ^2**		1.44	0.84	1.35	0.87	0.95	1.05

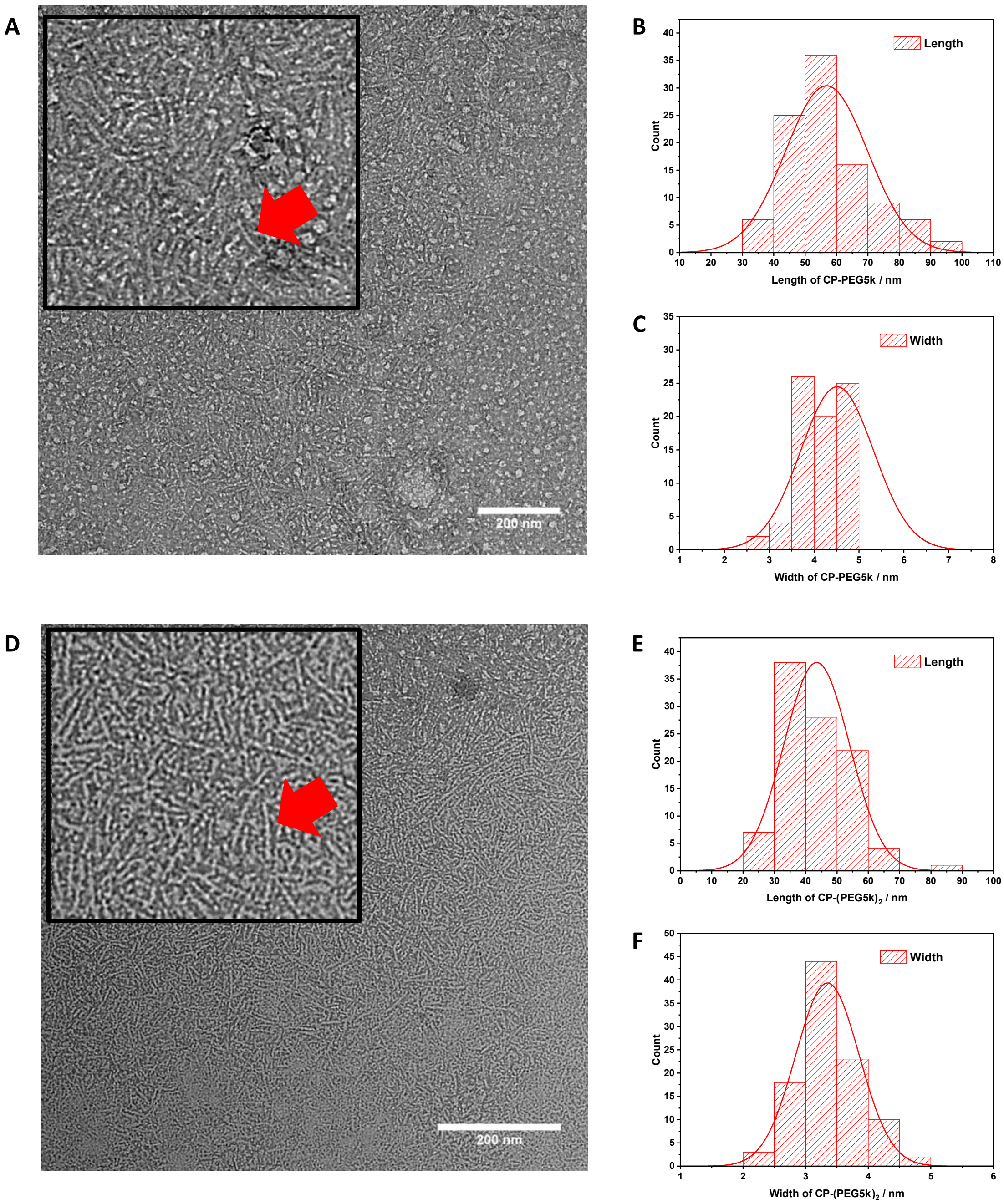

Figure S19 TEM image of one-arm CP-PEG5K (A) in pH7 and the distribution of length (B) and width (C) (stained with UOAc). TEM image of two-arm CP-(PEG5K)2 (D) in pH7 and the distribution of length (E) and width (F) (stained with UOAc).

TEM

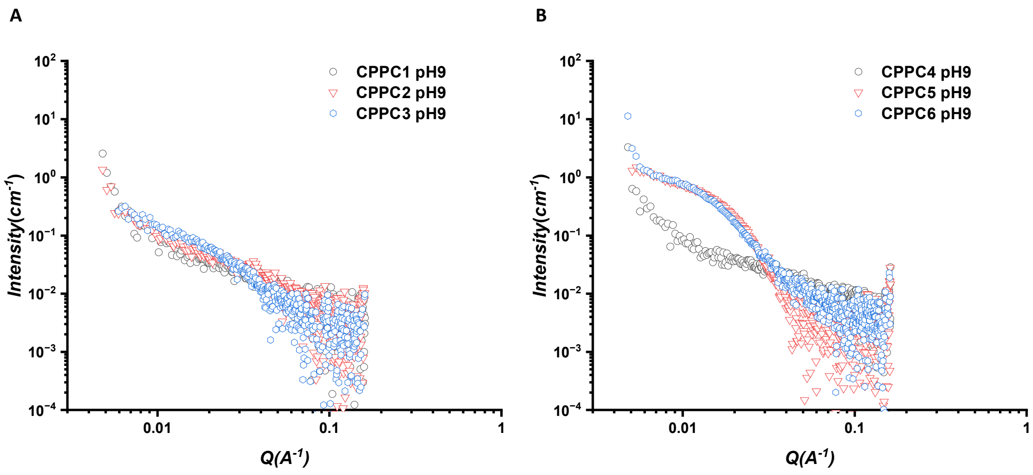

Figure S20 SAXS data for (A) pDMAEMA conjugates including pDMAEMA$_{11}$-CP-PEG5k (CPPC1), pDMAEMA$_{23}$-CP-PEG5k (CPPC2), pDMAEMA$_{23}$-CP-PEG10k (CPPC3) and (B) pDEAEMA conjugates including pDEAEMA$_{12}$-CP-PEG5k (CPPC4), pDEAEMA$_{24}$-CP-PEG5k (CPPC5), pDEAEMA$_{24}$-CP-PEG10k (CPPC6).

SAXS

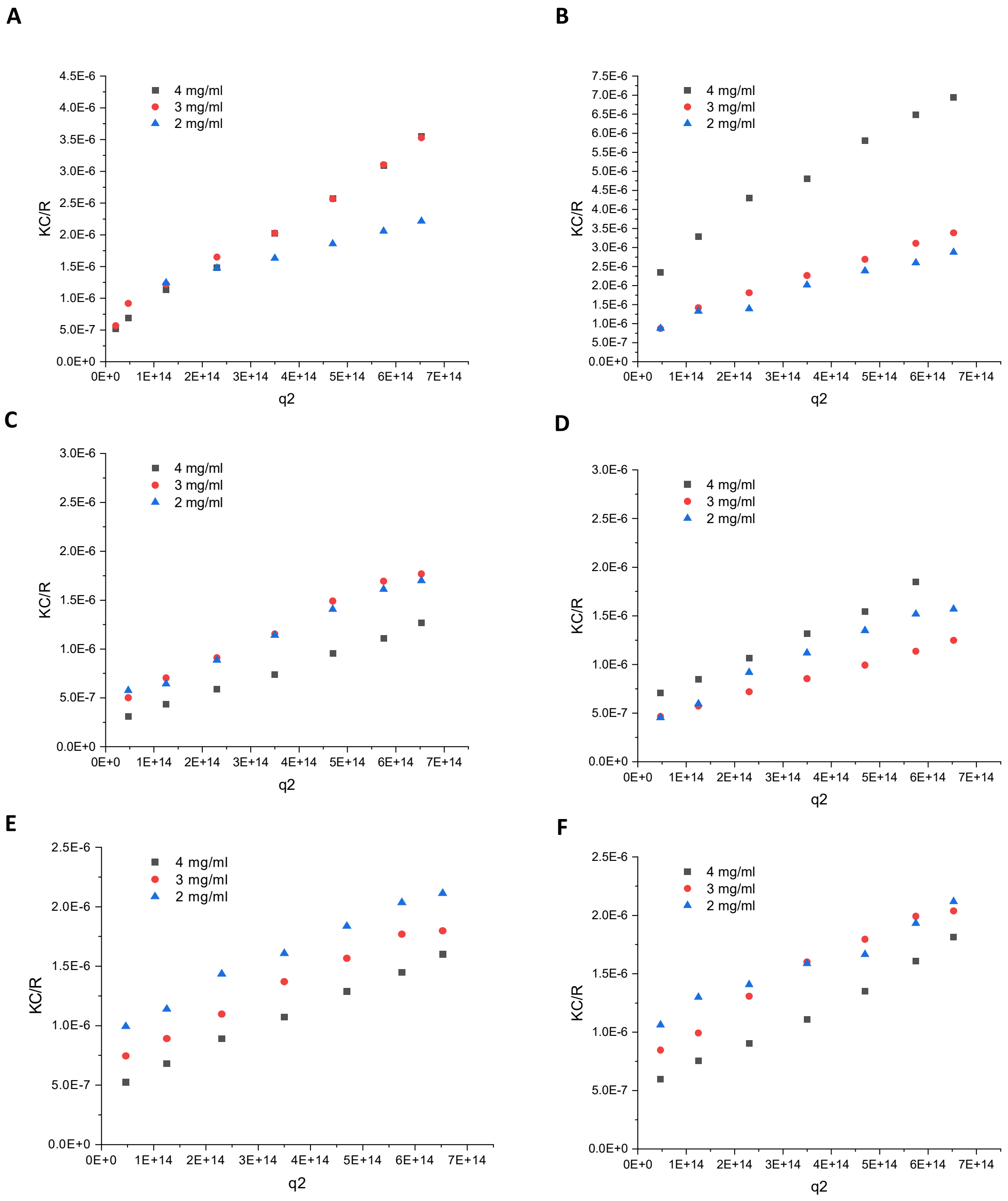

SLS data summary

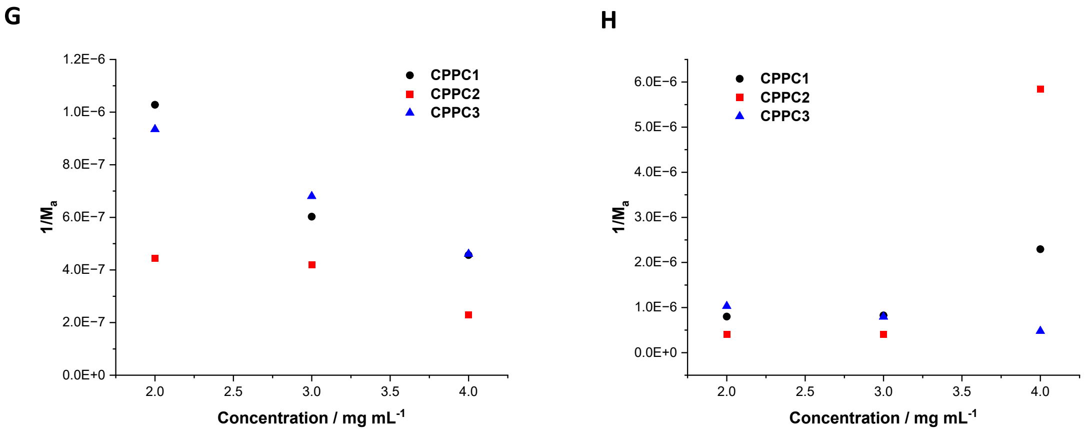

Figure S21 Evolution of KC/R of pDMAEMA conjugates as a function of q2 obtained by SLS, CPPC1 at pH9 (**A**) and pH3 (**B**), CPPC2 at pH9 (**C**) and pH3 (**D**), CPPC3 at pH9 (**E**) and pH3 (**F**) with different concentrations from 2.0 to 4.0 mg/mL. Evolution of 1/Ma of pDMAEMA conjugates as a function of concentration at pH9 (**G**) and pH3 (**H**) obtained by static light scattering.

Table S12 Data summary for pDMAEMA conjugates determined by SLS.

Sample	pH	Concentration/ (mg/mL)	M_a	N_{agg}	Length / nm
**CPPC1**	**pH9**	4	2.19E+06	261	123
		3	1.66E+06	197	93
		2	9.73E+05	116	55
	**pH3**	4	4.36E+05	55	26
		3	1.21E+06	153	72
		2	1.25E+06	159	75
**CPPC2**	**pH9**	4	4.34E+06	433	204
		3	2.38E+06	243	114
		2	2.25E+06	230	108
	**pH3**	4	1.71E+05	174	82
		3	2.43E+06	248	117
		2	2.45E+06	250	118
**CPPC3**	**pH9**	4	2.17E+06	146	69
		3	1.47E+06	99	47
		2	1.07E+06	72	34
	**pH3**	4	2.09E+06	141	66
		3	1.26E+06	85	40
		2	9.70E+05	66	31

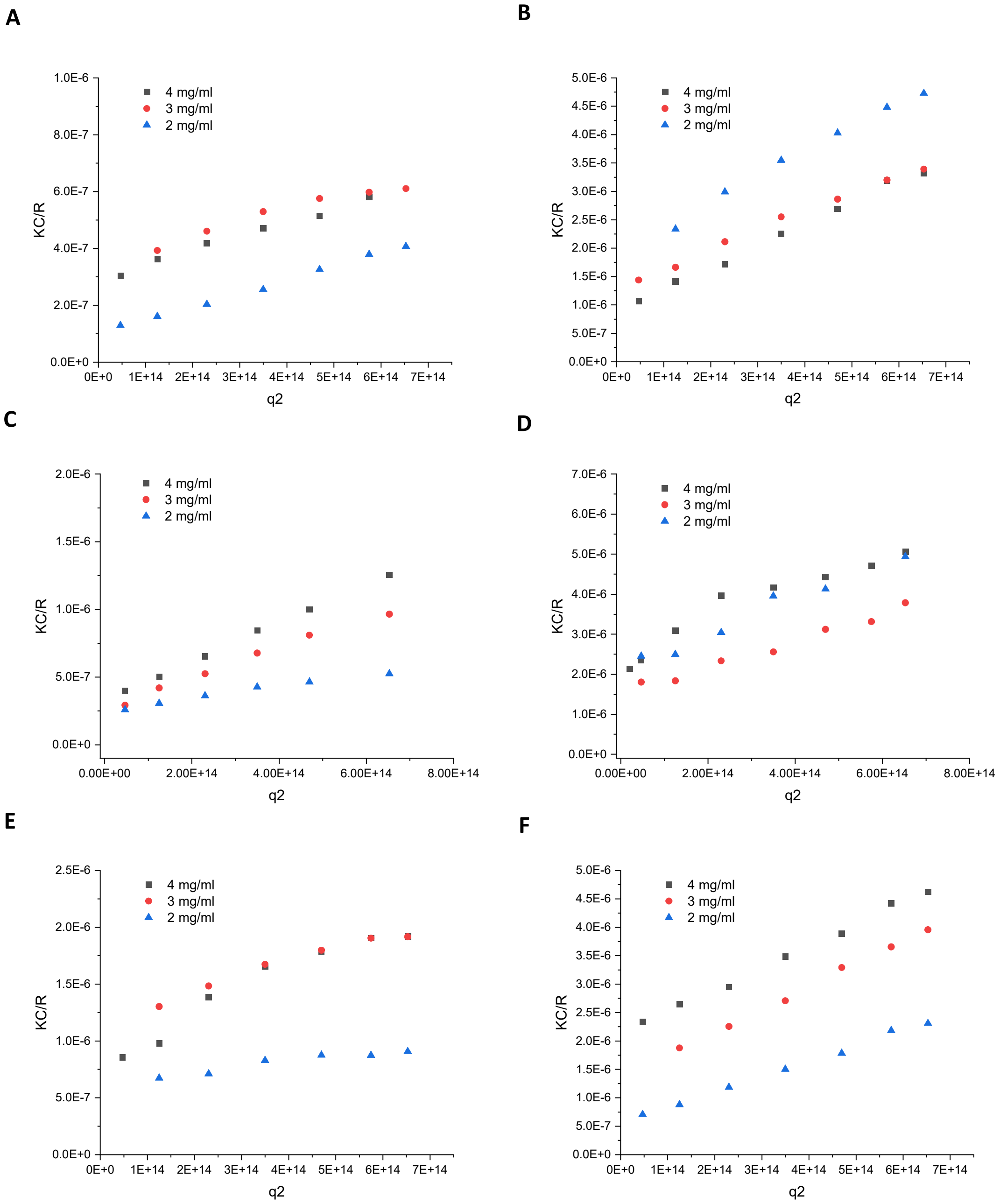
A
B
C
D
E
F
4 mg/ml
3 mg/ml
2 mg/ml
KC/R
q2

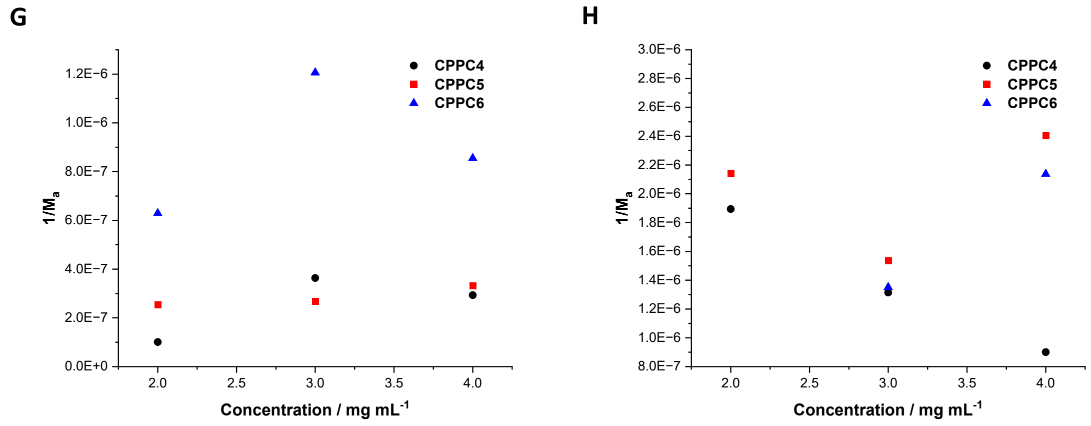

Figure S22 Evolution of KC/R of pDEAEMA conjugates as a function of q^2 obtained by SLS, CPPC4 at pH9 (**A**) and pH3 (**B**), CPPC5 at pH9 (**C**) and pH3 (**D**), CPPC6 at pH9 (**E**) and pH3 (**F**) with different concentrations from 2.0 to 4.0 mg/mL. Evolution of $1/M_a$ of pDEAEMA conjugates as a function of concentration at pH9 (**G**) and pH3 (**H**) obtained by static light scattering.

Table S13 Data summary for pDEAEMA conjugates determined by SLS.

Sample	pH	Concentration/ (mg/mL)	M_a	N_{agg}	Length / nm
CPPC4	pH9	4	3.41E+06	406	191
		3	2.75E+06	328	154
		2	2.09E+06	249	117
	pH3	4	1.11E+06	132	62
		3	7.61E+05	91	43
		2	5.28E+05	63	30
CPPC5	pH9	4	3.02E+06	280	132
		3	3.72E+06	344	162
		2	3.95E+06	365	172
	pH3	4	4.16E+05	38	18
		3	6.52E+05	60	28
		2	4.67E+05	43	20
CPPC6	pH9	4	1.17E+06	74	35
		3	8.29E+05	53	25
		2	1.59E+06	100	47
	pH3	4	4.68E+05	30	14
		3	7.40E+05	47	22
		2	1.79E+06	113	53

References

1. Arnold, O.; Bilheux, J. C.; Borreguero, J. M.; Buts, A.; Campbell, S. I.; Chapon, L.; Doucet, M.; Draper, N.; Ferraz Leal, R.; Gigg, M. A.; Lynch, V. E.; Markvardsen, A.; Mikkelson, D. J.; Mikkelson, R. L.; Miller, R.; Palmen, K.; Parker, P.; Passos, G.; Perring, T. G.; Peterson, P. F.; Ren, S.; Reuter, M. A.; Savici, A. T.; Taylor, J. W.; Taylor, R. J.; Tolchenov, R.; Zhou, W.; Zikovsky, J., Mantid—Data analysis and visualization package for neutron scattering and μ SR experiments. *Nuclear Instruments and Methods in Physics Research Section A: Accelerators, Spectrometers, Detectors and Associated Equipment* **2014,** *764*, 156-166.
